# Hydroxysteroid 17-β dehydrogenase 14 (HSD17B14) is an L-fucose dehydrogenase, the initial enzyme of the L-fucose degradation pathway

**DOI:** 10.1016/j.jbc.2024.107501

**Published:** 2024-06-27

**Authors:** Apolonia Witecka, Varvara Kazak, Sebastian Kwiatkowski, Anna Kiersztan, Adam K. Jagielski, Wiktor Kozminski, Rafal Augustyniak, Jakub Drozak

**Affiliations:** 1 Department of Metabolic Regulation, Institute of Biochemistry, Faculty of Biology, University of Warsaw, Warsaw, Poland; 2 Biotechnology Division, Research & Development Centre, Celon Pharma S.A., Kazun Nowy, Poland; 3 Biological and Chemical Research Centre, Faculty of Chemistry, University of Warsaw, Warsaw, Poland

**Keywords:** L-fucose, L-arabinose, SDR superfamily, HSD17B14, DHRS10, retSDR3, L-fucose dehydrogenase

## Abstract

L-Fucose (6-deoxy-L-galactose), a monosaccharide abundant in glycolipids and glycoproteins produced by mammalian cells, has been extensively studied for its role in intracellular biosynthesis and recycling of GDP-L-fucose for fucosylation. However, in certain mammalian species, L-fucose is efficiently broken down to pyruvate and lactate in a poorly understood metabolic pathway. In the 1970s, L-fucose dehydrogenase, an enzyme responsible for the initial step of this pathway, was partially purified from pig and rabbit livers and characterized biochemically. However, its molecular identity remained elusive until recently. This study reports the purification, identification, and biochemical characterization of the mammalian L-fucose dehydrogenase. The enzyme was purified from rabbit liver approximately 340-fold. Mass spectrometry analysis of the purified protein preparation identified mammalian hydroxysteroid 17-β dehydrogenase 14 (HSD17B14) as the sole candidate enzyme. Rabbit and human HSD17B14 were expressed in HEK293T and *Escherichia coli*, respectively, purified, and demonstrated to catalyze the oxidation of L-fucose to L-fucono-1,5-lactone, as confirmed by mass spectrometry and NMR analysis. Substrate specificity studies revealed that L-fucose is the preferred substrate for both enzymes. The human enzyme exhibited a catalytic efficiency for L-fucose that was 359-fold higher than its efficiency for estradiol. Additionally, recombinant rat HSD17B14 exhibited negligible activity towards L-fucose, consistent with the absence of L-fucose metabolism in this species. The identification of the gene-encoding mammalian L-fucose dehydrogenase provides novel insights into the substrate specificity of enzymes belonging to the 17-β-hydroxysteroid dehydrogenase family. This discovery also paves the way for unraveling the physiological functions of the L-fucose degradation pathway, which remains enigmatic.

L-Fucose (6-deoxy-L-galactose) is a monosaccharide abundantly present in mammals. Unlike all other endogenous hexoses, it exhibits L-configuration and is devoid of hydroxyl group on the last carbon atom (C6) (Fig. 1). The monosaccharide is a common component of glycolipids and glycoproteins produced by mammalian cells, where it is present as a terminal modification of *N*- and *O*-linked glycans, decorates the core of complex glycans, or is directly linked to serine or threonine residues of proteins, containing epidermal growth factor–like repeats or thrombospondin type 1 repeats [for review, see (1)].

**Figure 1:**
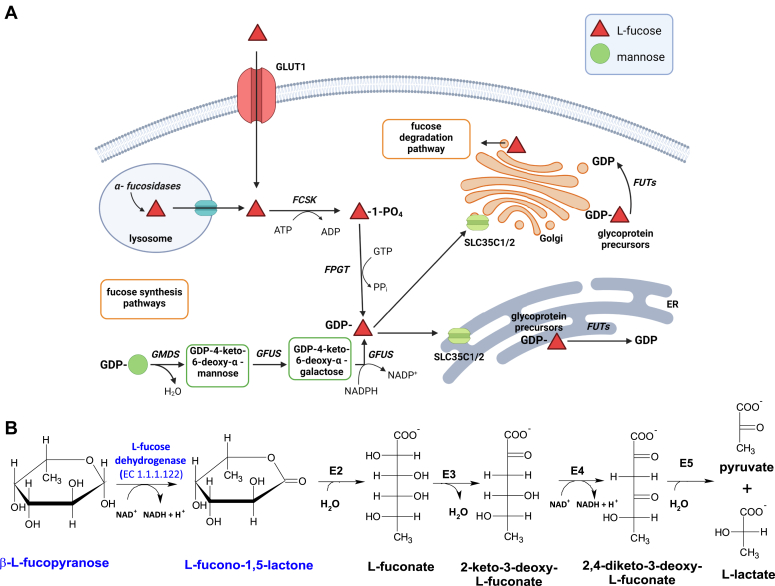
**Intracellular metabolism of L-fucose in mammals**. *A*, biosynthesis of GDP-L-fucose. GDP-L-fucose is formed through two independent cytosolic pathways. The *de novo* pathway present in most mammalian tissues converts GDP-D-mannose to GDP-L-fucose through three enzymatic reactions catalyzed by two enzymes—GDP-D-mannose 4,6-dehydratase (GMDS) and bifunctional GDP-L-fucose synthase (GFUS). An alternative salvage pathway is present mostly in the liver and kidney of mammals and generates GDP-L-fucose from free L-fucose. The monosaccharide derives from extracellular spaces or can be liberated from fucosylated glycans by lysosomal α-fucosidases. GLUT 1 facilitates the intracellular uptake of L-fucose, while the mechanism of L-fucose transport from the lysosomal lumen to the cytosol remains unknown. The salvage pathway is initiated by the phosphorylation of L-fucose to L-fucose-1-phosphate. This reaction is catalyzed by fucokinase (FCSK). Subsequently, fucose-1-phosphate guanylyltransferase (FPGT) utilizes L-fucose-1-phosphate and GTP as substrates to form GDP-L-fucose, releasing pyrophosphate as a byproduct. GDP-L-fucose synthesized by these pathways is transported into the lumen of the endoplasmic reticulum and the Golgi apparatus (*via* SLC35C1 and SLC35C2 transporters) where it supplies the catalytic domains of fucosyltransferases. This figure was created with BioRender.com. *B*, degradation of L-fucose. In the liver and kidney of certain mammals, L-fucose is broken down to pyruvate and L-lactate. The pathway is initiated by L-fucose dehydrogenase that catalyzes the oxidation of monosaccharide to L-fucono-1,5-lactone, which has been reported to spontaneously hydrolyze to L-fuconate. Alternatively, lactone hydrolysis might be catalyzed by a specific lactonase (E2), as such an enzyme operates in the L-fucose degradation pathway in bacteria. Next, L-fuconate dehydratase (E3) converts L-fuconate to 2-keto-3-deoxy-L-fuconate that is subsequently oxidized to 2,4-diketo-3-deoxy-L-fuconate in reaction catalyzed by the NAD^+^-dependent dehydrogenase (E4). Finally, 2,4-diketo-3-deoxy-L-fuconate hydrolase (E5) completes the pathway, yielding pyruvate and L-lactate, which can be further oxidized to CO_2_ in energy metabolism. Biological relevance of the pathway remains unexplored.

L-Fucose is certainly best known as a constituent of H-antigen that determines the O blood type and forms the core glycan of A and B epitopes [for review, see (2)]. However, the biological importance of this monosaccharide in mammals is far more complex as fucosylated glycans contribute in regulating a wide range of processes, including immune response, signal transduction, and cell adhesion (1). For example, L-fucose is an essential component of carbohydrate ligands presented by endothelial cells lining venules. These extracellular glycans are recognized by L-selectins expressed by leukocytes. Interaction between selectins and their ligands enables the rolling of leukocytes on the endothelium that is the first step in leukocyte extravasation (3). In humans, the loss of L-fucose residues in selectin ligands due to pathogenic mutations in GDP-fucose transporter (SLC35C1) localized in Golgi apparatus results in leukocyte adhesion deficiency type II (OMIM 266265), a congenital disorder of glycosylation that is characterized by immunodeficiency, mental, and growth retardation (4). Furthermore, cell-surface–fucosylated antigens are frequently employed by cancer cells to promote their migration and metastasis [for review, see (5)]. They are upregulated in a variety of tumor types, including lung, breast, and colorectal cancers. Interestingly, the elimination of the terminal L-fucose from these antigens was shown to impair the ability of cancer cells to roll within endothelial tissue, retarding their invasive capability (6).

In mammalian cells, fucosylation takes place mostly in Golgi apparatus where at least 11 membrane-bound fucosylotransferases (FUTs) catalyze fucosylation of *N*- and *O*-glycans and glycolipids (Fig. 1*A*). There are also two distinct protein *O*-fucosylotransferases residing in the endoplasmic reticulum that add L-fucose directly to Ser/Thr residues of proteins. FUTs utilize GDP-fucose as a donor of L-fucosyl moiety, whereas SLC35C1 and SLC35C2 proteins are GDP-fucose transporters facilitating the transfer of the active form of L-fucose from cytosol to Golgi and endoplasmic reticulum lumen (2). The intracellular formation of GDP-L-fucose is achieved *via* two independent cytosolic pathways, *de novo* synthesis or salvage/recycling of L-fucose (*cf.* Fig. 1*A*). The *de novo* pathway transforms GDP-D-mannose to GDP-L-fucose through three enzymatic reactions catalyzed by two enzymes—GDP-D-mannose 4,6-dehydratase and bifunctional GDP-L-fucose synthase, whereas the fucose salvage pathway requires the activity of fucokinase (FCSK) and fucose-1-phosphate guanylyltransferase to generate GDP-D-fucose from free L-fucose derived from extracellular or lysosomal sources. Yet, virtually nothing is known about the mechanism behind the transport of L-fucose from the lysosomal lumen to the cytosol, whereas plasma membrane transporter GLUT1 has been recently identified as a highly efficient L-fucose transporter (7). Also, the biochemical importance of the existence of two distinct pathways for the biosynthesis of GDP-L-fucose in mammalian cells remains largely unclear. However, recent data suggests that these two pathways plausibly provide GDP-L-fucose to distinct, independent, and separate cytosolic pools of this compound that serve to supply different FUTs (8, 9).

During the past 60 years, a lot of effort has been made to disclose the biochemistry and physiological importance of L-fucose in mammals; however, surprisingly little is still known about the catabolism of this monosaccharide. Early studies with human subjects showed that following the intravenous administration of radiolabeled L-fucose, ≈40% of this monosaccharide is readily oxidized to ^14^CO_2_, indicating that it could be metabolized to smaller metabolic units (10). Furthermore, after oral administration of fucose to healthy volunteers (100 mg/kg body weight), the monosaccharide was rapidly cleared from the serum with a half time of 100 min (11). Similar findings were also reported for cats, Guinea pigs, and rabbits (12). These *in vivo* observations were further supported by results showing that in pork liver and kidney, L-fucose is effectively broken down to pyruvate and L-lactate in a metabolic pathway that resembles that one present in some bacterial species (*cf.* Fig. 1*B*) (13). This pathway is initiated by L-fucose dehydrogenase that catalyzes the NAD^+^-dependent oxidation of the monosaccharide to L-fucono-1,5-lactone, an unstable compound, spontaneously hydrolyzing to L-fuconate (14). However, it cannot be excluded that lactone hydrolysis is in fact catalyzed by a specific lactonase, as such an enzyme operates in the bacterial L-fucose degradation pathway (15). Next, L-fuconate dehydratase converts L-fuconate to 2-keto-3-deoxy-L-fuconate that is subsequently oxidized to 2,4-diketo-3-deoxy-L-fuconate in the reaction catalyzed by the NAD^+^-dependent dehydrogenase. Finally, 2,4-diketo-3-deoxy-L-fuconate hydrolase completes the pathway, yielding pyruvate and L-lactate, which can be further oxidized to CO_2_ in energy metabolism.

The porcine enzymes contributing to the degradation of L-fucose were partially purified and characterized to varying degrees by Schachter *et al.* in 1970s (13, 14, 16, 17). Later, L-fucose dehydrogenase from rabbit and sheep liver was also purified and studied in more detail by others (18, 19). However, L-fuconate dehydratase identified as enolase superfamily member 1 has been remaining the only known mammalian enzyme of this pathway so far (20). Recently, FAHD1 protein has been suggested to act as 2,4-diketo-3-deoxy-L-fuconate hydrolase in human cells (21), while the molecular identities and biochemical properties of all other enzymes of that pathway need to be disclosed. Also, the physiological role of L-fucose degradation has yet to be estimated in cells, tissues, and organisms of mammals.

Here, we report the identification of mammalian L-fucose dehydrogenase (EC 1.1.1.122) as hydroxysteroid 17-β dehydrogenase 14 (HSD17B14, DHRS10), which was previously shown to oxidize estradiol and testosterone at the C-17 position (22). We characterized this enzyme biochemically and showed that it catalyzes the conversion of L-fucose to L-fucono-1,5-lactone, with favored substrate specificity and catalytic efficiency. Therefore, HSD17B14 is more likely the initial enzyme in the postulated L-fucose degradation pathway than the enzyme involved in the metabolism of steroids in mammals. This molecular identification substantially expands our understanding of substrate specificity within the 17-β-hydroxysteroid dehydrogenase family. Previously, all members were believed to exclusively metabolize lipophilic compounds like steroids, fatty acids, and bile acids (23). This work also highlights the need for further investigation into the, as yet, unclear, biochemical, and physiological role of L-fucose breakdown.

## Results

### Purification and identification of rabbit L-fucose dehydrogenase

**Figure 2:**
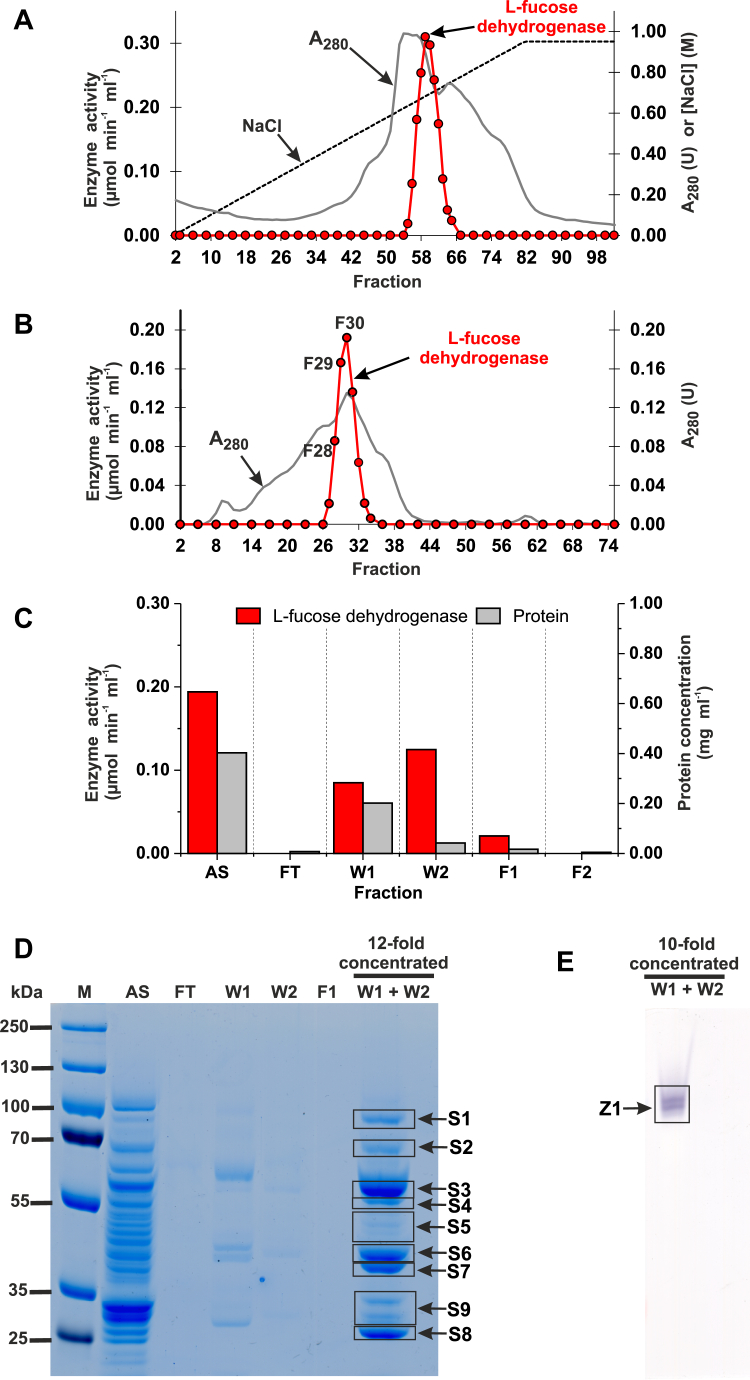
**Purification of the rabbit L-fucose dehydrogenase.** The enzyme was purified by column chromatography on (*A*) DEAE Sepharose, (*B*) Superdex 200 16/60 HiLoad, and (*C*) Reactive Red 120 Agarose, as described in the "Experimental procedures" section. Resulted fractions were tested for the activity of L-fucose dehydrogenase. *D*, the indicated fractions from the Reactive Red 120 Agarose column were analyzed by SDS-PAGE, and the gel was stained with PageBlue protein staining solution (Thermo Fisher Scientific). In addition, fractions W1 and W2 were concentrated 12-fold by ultrafiltration (Vivaspin 500) before being analyzed. *E*, also, 10-fold concentrated fractions W1 and W2 were analyzed by native PAGE and subsequently stained for L-fucose dehydrogenase activity (zymography). All indicated bands were cut out from the gel and analyzed by mass spectrometry. Fractions F1 and F2 were eluted with 300 mM NaCl. M, prestained protein marker; AS, applied sample (Fraction 31 from Superdex 200); FT, flow through; W1 and W2, wash one and two, respectively.

At all stages of the purification process, L-fucose dehydrogenase activity was monitored spectrophotometrically by measuring the rate of the NAD^+^ (λ = 340 mn) reduction in the presence of L-fucose. The enzyme was purified from rabbit liver about 340-fold by a six-step process involving fractionation with PEG (PEG4000), ion-exchange chromatography on DEAE-Sepharose resin, dialysis to sodium phosphate buffer, pH = 5.0, salting-out with 3M NaCl, gel filtration on Superdex 200 column, and affinity chromatography on Reactive Red 120 Agarose column (Fig. 2, *A*–*C*). Enzyme activity corresponded with one peak throughout the purification process. The yield of the purification was 0.5% based on total recovered activity (Table 1).

**Table 1:** Purification of L-fucose dehydrogenase from rabbit liver

Fraction	Volume	Total protein	Total activity	Specific activity	Purification	Yield
	mL	Mg	μmol.min^-1^	μmol.min^-1^.mg^-1^	X-fold	%
Homogenate (Supernatant)^a^	195.0	ND	ND	ND	ND	ND
PEG 4000 (30–50%)	180.0	6244.7	17.17	0.003	1	100.0
DEAE Sepharose	28.0	831.3	7.72	0.009	3	40.5
pH = 5.0 (Supernatant)	10.0	113.0	2.33	0.021	8	13.6
3M NaCl	2.5	26.0	1.39	0.053	19	8.1
Superdex 200 16/60 HiLoad	9.0	6.8	1.00	0.147	54	5.8
Reactive Red 120^b^	2.0	0.1	0.09	0.926	337	0.5

ND, not determined.

a The enzyme activity was undetectable in the homogenate and was measurable only after homogenate fractionation with PEG 4000.

b The data represent values only for the most purified fraction.

**Figure 3:**
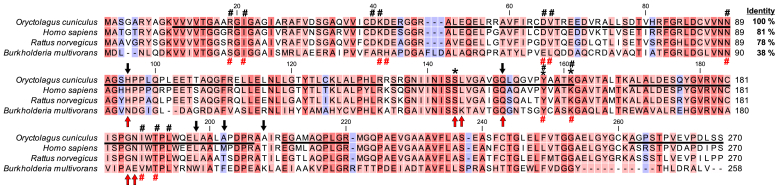
**Amino acid sequence alignment of rabbit HSD17B14 protein with its mammalian orthologs and bacterial L-fucose dehydrogenase.** The sequence of rabbit (*Oryctolagus cuniculus*) protein was retrieved from a translated RNA sequence (GBCH01173099.1) deposited in the transcriptome shotgun assembly (TSA) database, whereas sequences of human (*Homo sapiens*, NP_057330.2), rat (*Rattus norvegicus,* NP_001178040.1), and bacterial protein (*Burkholderia multivorans*, A0A0H3KNE7) were obtained from the NCBI Protein database. The percentage of amino acid identities with rabbit HSD17B14 protein is given in the *upper right*. Amino acid residues of human enzyme interacting with NAD(H) are indicated by *black hashes*; *black arrows* show residues of the active site–coordinating estrone—the postulated product of the catalyzed reaction, whereas *black asterisks* mark the key catalytic residues of human enzyme (31). By analogy, *red hashes* indicate amino acid residues interacting with NADP(H) in the bacterial L-fucose dehydrogenase, whereas *red arrows* show residues binding L-fucose (15). The level of residues conservation is indicated by the color of the background, and the warmer color the higher conservation. The peptides identified by mass spectrometry in the protein purified from rabbit liver are underlined in the rabbit sequence, and several peptides covering similar though shorter sequences have been omitted.

**Figure 4:**
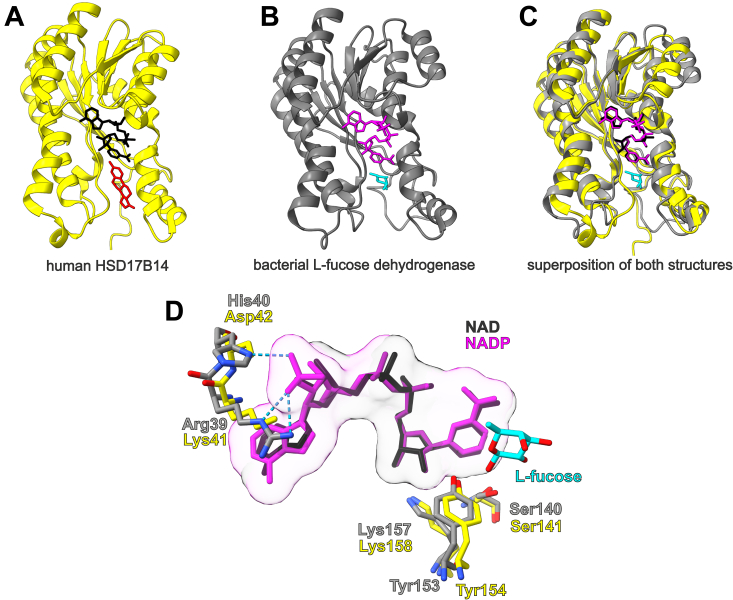
**Structural similarity between the human HSD17B14 protein and bacterial L-fucose dehydrogenase.** Ribbon representations of (*A*) human HSD17B14 (PDB: 5hs6), (*B*) *Burkholderia multivorans* L-fucose dehydrogenase (PDB: 4gvx), and (*C*) the superposition of both enzyme structures, highlighting a clearly similar fold architecture (a root mean square deviation of 2.43 Å). *A*, HSD17B14 is shown in complex with NAD (*black sticks*) and estrone (*red sticks*), whereas (*B*) L-fucose dehydrogenase is illustrated with bound NADP (*magenta sticks*) and L-fucose (*cyan sticks*). *C*, the estrone structure is omitted in the superposition for simplifying. *D*, close-up of the active sites of HSD17B14 and L-fucose dehydrogenase. Dinucleotide coenzymes superimpose very well, with a clear exception for the 2′-phosphate of NADP that is absent in NAD. In bacterial L-fucose dehydrogenase, the His40 residue plausibly facilitates the preferential biding of NADP over NAD, while the Asp42 residue present in HSD17B14 determines the enzyme preference for NAD. The catalytic triads consisting of Ser 140/141, Tyr153/154, and Lys 157/158 are also shown. All models were prepared using UCSF ChimeraX (46).

After its purification, the enzyme was concentrated about 10-fold and analyzed using SDS-PAGE and zymography. The later method allowed to visualize protein bands specifically corresponding to the dehydrogenase. SDS-PAGE analysis revealed nine polypeptides between about 25 to 100 kDa (*cf.* Fig. 2*D*) that coeluted with the enzyme activity in the fractions derived from the Reactive Red 120 Agarose purification step, while zymography indicated a double band species (*cf.* Fig. 2*E*). All bands were excised from the gels, digested with trypsin, and the resulting peptides were analyzed by tandem mass spectrometry (MS; quadrupole time of flight [Q-TOF]). The sequences of the identified peptides were then compared with the rabbit reference proteome from the National Center for Biotechnology Information (NCBI) protein database. Surprisingly, at first glance, the MS sequence data indicated no reasonable candidate for the L-fucose dehydrogenase. However, a detailed analysis of the rabbit and human proteomes revealed the presence of a fusion rabbit protein composed of HSD17B14 and a mitochondrial branched-chain-amino-acid aminotransferase, which were hidden under the name branched-chain-amino-acid aminotransferase, mitochondrial isoform X1 (NCBI Protein: XP_008250588.1). Since in human and other mammalian proteomes, these two proteins are always separate entities and their genes are adjacent to each other on chromosomes, we assumed that the fusion was only apparent and resulted from a mistake of automated sequencing data analysis. Furthermore, neither rabbit *HSD17B14* gene nor HSD17B14 protein were present in dedicated NCBI databases at the time of this analysis (Oct. 2021). However, we found a sequence GBCH01173099.1 (GenBank) in Transcriptome Shotgun Assembly Sequence Database (NCBI TSA) that encodes a rabbit protein homologous to human HSD17B14. Then, we reanalyzed the mass spectrometry data obtained with purified rabbit L-fucose dehydrogenase by comparing them with a rabbit proteome in which we had introduced the rabbit HSD17B14 sequence. This analysis revealed that HSD17B14 was the best hit for the 26-kDa polypeptide (Table S1). Twenty-nine matching peptides (underlined in Fig. 3) were found that covered more than 75% of the retrieved sequence. Importantly, HSD17B14 was also the most abundant protein and the only dehydrogenase present in protein bands visualized by zymography (*cf.* Table S1). These findings led us to hypothesize that HSD17B14 protein might be a functional L-fucose dehydrogenase in mammals. This conclusion was also supported by the following observations: (*i*) a monomeric molecular weight of pig liver L-fucose dehydrogenase was about 30 kDa, based on SDS-PAGE analysis (24), which is close to that value for rabbit HSD17B14 (≈28 kDa) and (*ii*) human HSD17B14 shares remarkable structural similarity with bacterial L-fucose dehydrogenase (Fig. 4, *A*–*C*).

### Rabbit, human, and rat HSD17B14 catalyze the oxidation of L-fucose in the presence of NAD^+^

**Figure 5:**
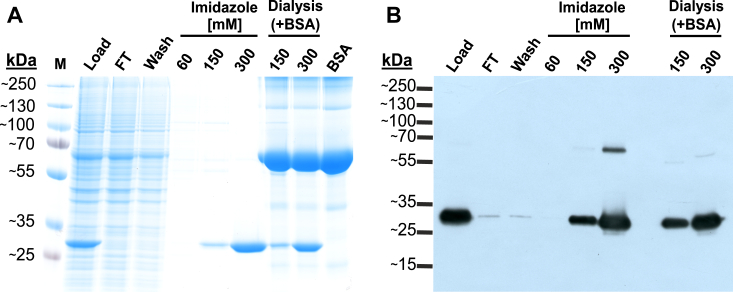
**SDS-PAGE and Western blot analysis of recombinant rabbit HSD17B14 purification**. Lysate of HEK-293T cells overexpressing the recombinant enzyme (LOAD) was applied to the HisTrap Crude column, and flow-through (FT) was collected. The column was washed with a buffer without imidazole (Wash). Retained proteins were eluted by applying the buffer with indicated concentrations of imidazole. To remove imidazole, 150-mM and 300-mM fractions were subjected to dialysis. Prior to the dialysis, fractions were supplemented with BSA (1 mg/ml) to improve the stability of purified enzyme. The purification process was analyzed by SDS-PAGE (*A*), whereas the presence of recombinant rbHSD17B14 protein was verified by Western blot analysis (*B*) using an antibody against the His_6_ tag. Analogous results were obtained for human and rat enzymes. Note that the 65 kDa band visible on Western blot likely corresponds to a dimer of the recombinant enzyme, suggesting incomplete denaturation during sample preparation. In dialyzed enzyme preparations, this band disappears, possibly due to the masking effect of BSA. The purity of both recombinant proteins was above 95%. M, prestained protein ladder.

**Figure 6:**
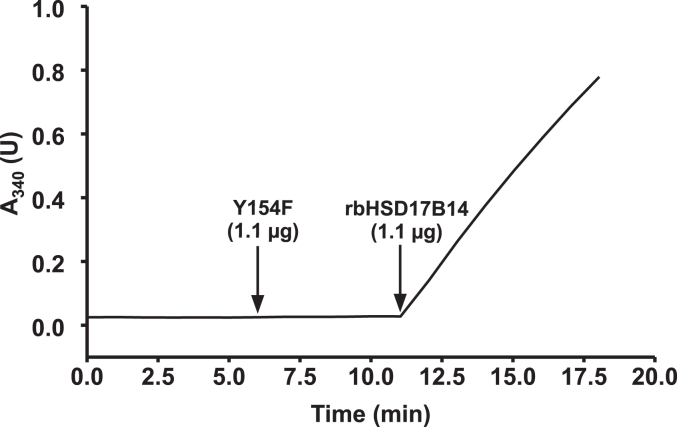
**Test of the enzymatic activity of the purified recombinant WT (rbHSD17B14) and mutated (Y154F) rabbit HSD17B14.** The enzyme activity was followed spectrophotometrically by measuring the conversion of NAD^+^ into NADH (λ = 340 nm). The reaction was performed with 1.1 μg of Y157F protein as described in the "Experimental procedures" section. The addition of 1.1 μg of WT rbHSD17B14 initiated L-fucose oxidation, concomitant with the reduction of NAD^+^ to NADH, as evidenced by the change in absorbance at 340 nm. The figure shows the results of a single representative assay. Human HSD17B14 showed similar enzymatic activity, while rat enzyme was less active. The results for human and rat enzymes as well as control reactions carried out in the absence of L-fucose are shown in Fig. S1.

To confirm the molecular identity of L-fucose dehydrogenase, rabbit HSD17B14 (rbHSD17B14) was expressed in HEK293T cells as a fusion protein with the *N*-terminal His_6_ tag. The recombinant enzyme was purified (Fig. 5) and shown to catalyze the oxidation of L-fucose in the presence of NAD^+^ (Figs. 6 and S1). To further confirm that the observed activity resulted from HSD17B14 and not from impurities that might hypothetically copurify with the recombinant proteins, the mutated form of rabbit enzyme was also produced (Y154F) and purified under the same conditions as the WT rbHSD17B14. The catalytic center of HSD17B14 consists of three amino acid residues: Ser141, Tyr154, and Lys158 (*cf.* Fig. 4*D*), which are highly conserved in all 17-β-HSDs. They are typical for most enzymes from the short-chain dehydrogenases/reductases family (25) and because of that, Y154 residue was chosen for mutagenic studies. This mutated form showed negligible activity with L-fucose and NAD^+^ as substrates compared to the WT rbHSD17B14 (*cf.* Fig. 6). Although rbHSD17B14 shares about 80% identity in its amino acid sequences with human HSD17B14 (hHSD17B14) and rat HSD17B14 (rHSD17B14) (*cf.* Fig. 3), implying similar enzymatic properties, the hHSD17B14 and rHSD17B14 were expressed in *Escherichia coli* to compare their activities. This was particularly interesting for rHSD17B14 due to the lack of substantial L-fucose catabolism in rats (12).

Human HSD17B14 was originally reported to be involved in NAD^+^-dependent oxidizing of estrogens, androgens, including estradiol, 5-androstene-3β,17β-diol, and testosterone (26). Notably, β-estradiol was postulated as a major substrate of the enzyme. To verify these findings, the oxidation rates for β-estradiol and L-fucose as substrates were compared in the reactions catalyzed by hHSD17B14, rHSD17B14, and rbHSD17B14. As shown in Fig. S2, in comparison to L-fucose, β-estradiol was an extremely poor substrate for HSD17B14. More precisely, the tested enzymes were about 50- to 2300-times more active toward 5 μM L-fucose (752.19 ± 9.67, 562.9 ± 72.95, and 132.95 ± 1.9 nmol × min^−1^ × mg^−1^ protein for human, rabbit, and rat HSD17B14, respectively) than to 5 μM β-estradiol (0.33 ± 0.58, 0.59 ± 0.16, and 2.56 ± 0.45 nmol × min^−1^ × mg^−1^ protein, for human, rabbit, and rat HSD17B14, respectively), indicating that β-estradiol is unlikely to be a physiological substrate for HSD17B14.

These results led us to verify the substrate specificity of HSD17B14 with more biologically relevant substrates, including D-threose, D-ribose, D-arabinose, D-lyxose, L-xylose, L- and D-fucose, L- and D-galactose. Out of all tested compounds, only L-fucose and, albeit to a lesser extent, D-arabinose, L-galactose, L-xylose, and D-lyxose were accepted as substrates by the enzymes. Importantly, the rat enzyme was substantially less active towards the sugars than rabbit and human HSD17B14, though it was the only enzyme showing an activity in the presence of D-threose (Table 2). These results suggest that other compounds, plausibly monosaccharides, are preferred substrates for the rat enzyme.

**Table 2:** Substrate specificity of the recombinant rabbit, human, and rat HSD17B14

Structure	Substrate	rbHSD17B14	hHSD17B14	rHSD17B14
		μmol min^−1^ mg^−1^	μmol min^−1^ mg^−1^	μmol min^−1^ mg^−1^
	2 mM L-fucose	16.24 ± 1.16	29.63±0.61	0.61±0.04
	2 mM D-fucose 20 mM D-fucose	ND ND	ND ND	ND ND
	2 mM D-arabinose	10.73 ± 0.61	16.77 ± 1.16	0.47 ± 0.03
	2 mM L-galactose	3.05 ± 0.18	4.14 ± 0.39	0.35 ± 0.01
	2 mM D-galactose 20 mM D-galactose	ND ND	ND ND	ND ND
	2 mM L-xylose	0.42 ± 0.05	0.29 ± 0.01	0.16 ± 0.01
	2 mM D-lyxose	0.25 ± 0.02	0.18 ± 0.00	0.09 ± 0.00
	2 mM D-ribose 20 mM D-ribose	ND ND	ND ND	ND 0.08 ± 0.03
	2 mM D-threose	ND	ND	0.21 ± 0.01

Activity assays were performed spectrophotometrically (hHSD17B14 and rbHSD17B14) or fluorometrically (rHSD17B14) with the use of 1.1 to 5.0 μg of recombinant HSD17B14 proteins in the presence of 0.5 mM NAD^+^ and indicated concentration of the potential substrate. Values are the means ± SD (error bars) of three independent experiments. ND, not detected.

**Figure 7:**
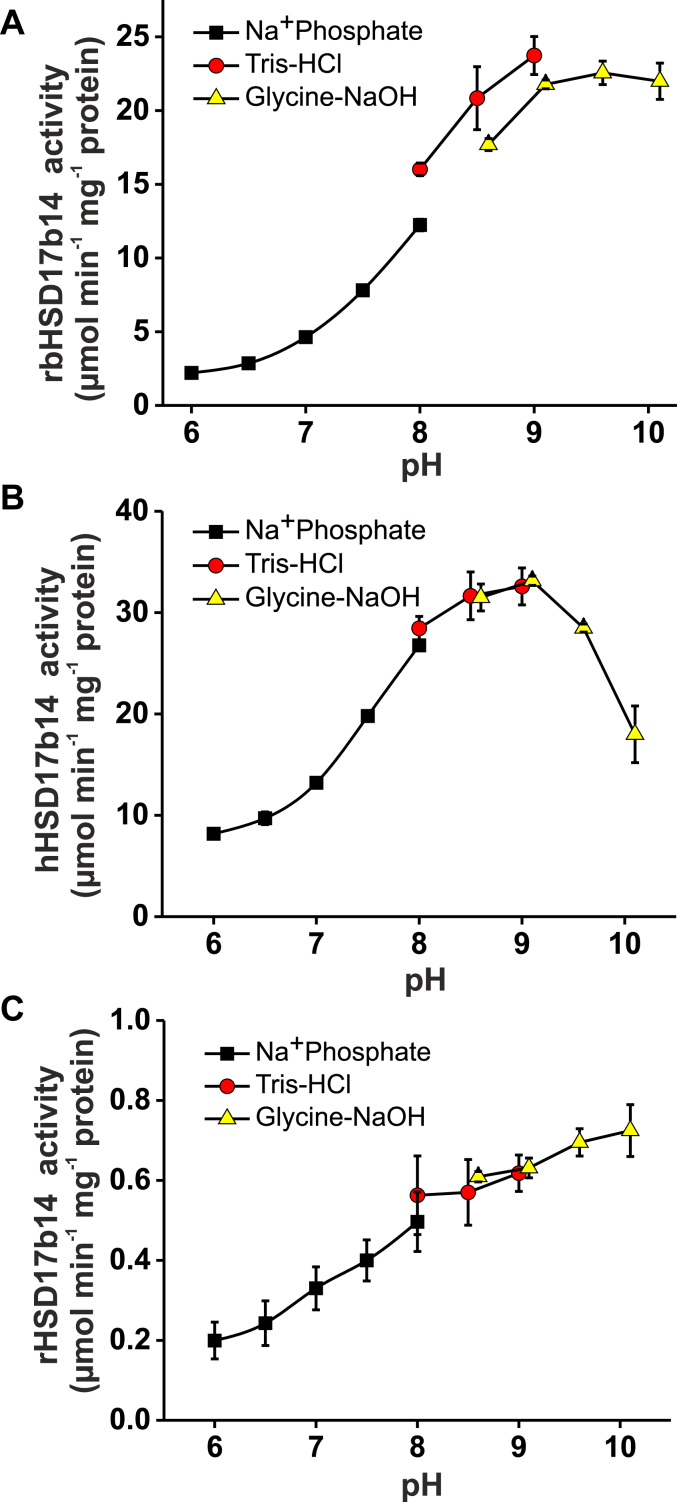
**Effect of the pH on the activity of the recombinant rabbit, human, and rat HSD17B14.** The activity of (*A*) rabbit, (*B*) human, and (*C*) rat enzyme was followed spectrophotometrically by measuring the conversion of NAD^+^ into NADH (λ = 340 nm). The reaction was performed as described in the "Experimental procedures". Sodium phosphate buffer was used for the lower pH values, Tris-HCl for higher pH values, whereas glycine-sodium hydroxide buffer for the most alkaline conditions. Values are the means ± SD (error bars) of three independent experiments. When an error bar is not visible, the error is smaller than the width of the line.

It was previously reported that the optimum pH value for the reaction catalyzed by L-fucose dehydrogenase is about 8.7 (14) or 10 (18). To verify these information, we determined the pH range of the dehydrogenase activity (Fig. 7). The obtained results were consistent with the literature data. All three enzymes showed the highest catalytic activity in a narrow pH range. Interestingly, the human enzyme exhibited the highest activity in the pH range from 8.5 to 9.0, with a decrease at higher pH values, whereas the rabbit and rat enzymes were still active at pH 10. Notably, the activity of rHSD17B14 was at a similar level in a broader pH range than rbHSD17B14 and hHSD17B14. Moreover, in lower pH ranges (6.0–7.5), all three enzymes are much less active. These results indicate that HSD17B14 is most likely the same L-fucose–oxidizing enzyme studied by Schachter *et al.* (14) and Endo and Hiyama (18).

The kinetic properties of the recombinant HSD17B14 proteins were investigated in detail using homogenous recombinant proteins and are compared in Table 3 and the analyses are shown in Fig. S3. All three enzymes followed the Michaelis–Menten model of enzyme kinetics. The K_M_ values for L-fucose obtained in our investigation for recombinant rabbit and human enzymes (K_M_ ≈ 0.136 mM and K_M_ ≈ 0.172 mM, respectively) were comparable to those determined for the partially purified rabbit enzyme (K_M_ ≈ 0.15 mM) (18) but about two times lower than the value determined for the pork liver L-fucose dehydrogenase (K_M_ ≈ 0.32 mM) (14). Also, the K_M_ value of rbHSD17B14 for NAD^+^ was 0.046 mM, which is lower than reported by Endo and Hiyama (18) (K_M_ ≈ 0.070 mM). The value for hHSD17B14 was relatively high (K_M_ ≈ 0.149 mM) (Fig. S3), though it lied in the physiological range of the intracellular NAD^+^ concentration reported in human cells (0.2–1 mM, (27)). As the K_M_ values of the recombinant rHSD17B14 could not be accurately determined in the spectrophotometric assay due to its limited sensitivity, they were fluorometrically estimated at ≈21 μM and ≈47 μM for L-fucose and NAD^+^, respectively (*cf.* Table 3 and Fig. S3). NADP^+^ was accepted as the coenzyme by neither of the HSD17B14 enzymes (data not shown).

**Table 3:** Kinetic properties of rbHSD17B14, hHSD17B14, and rHSD17B14 proteins

Substrate	rbHSD17B14			hHSD17B14			rHSD17B14		
	*V*_max_	*K*_M_	*k*_cat_^a^	*V*_max_	*K*_M_	*k*_cat_^a^	*V*_max_	*K*_M_	*k*_cat_^a^
	*μmol min*^*−1*^*mg*^*−1*^	*μM*	*s*^*−1*^	*μmol min*^*−1*^*mg*^*−1*^	*μM*	*s*^*−1*^	*μmol min*^*-1*^*mg*^*−1*^	*μM*	*s*^*−1*^
L-fucose	13.15 ± 0.22	136.36 ± 9.10	7.00 ± 0.12	27.20 ± 0.48	172.20 ± 11.40	14.00 ± 0.25	0.26 ± 0.01	21.45 ± 4.09	0.13 ± 0.01
NAD^+^	15.88 ± 0.19	45.69 ± 1.89	8.46 ± 0.10	32.82 ± 0.59	149.02 ± 8.13	16.89 ± 0.30	0.30 ± 0.01	47.06 ± 7.56	0.15 ± 0.01

Kinetic properties were determined with the use of purified recombinant *N*-terminal His_6_-tagged HSD17B14 proteins. Determinations for L-fucose were performed with 1.05 to 2.2 μg of the enzyme preparations in the presence of 1.5 mM NAD^+^ and variable concentrations of L-fucose, whereas the measurements for NAD^+^ were done in the presence of 2 mM L-fucose and variable concentrations of NAD^+^. Values are the means ± SD (error bars) of three independent experiments.

a Calculated for the His_6_-tagged recombinant enzymes with molecular weight =31,948 Da, 30,874 Da, and 30,314 Da for the rabbit, human, and rat enzyme, respectively.

Based on catalytic efficiency (*k*_*cat*_/K_M_), L-fucose was about 8 and 13 times better substrate for rabbit and human dehydrogenase than for the rat enzyme, again indicating that the rat enzyme prefers other compounds.

### Evidence for L-fucono-1,5-lactone as the product of HSD17B14 activity

**Figure 8:**
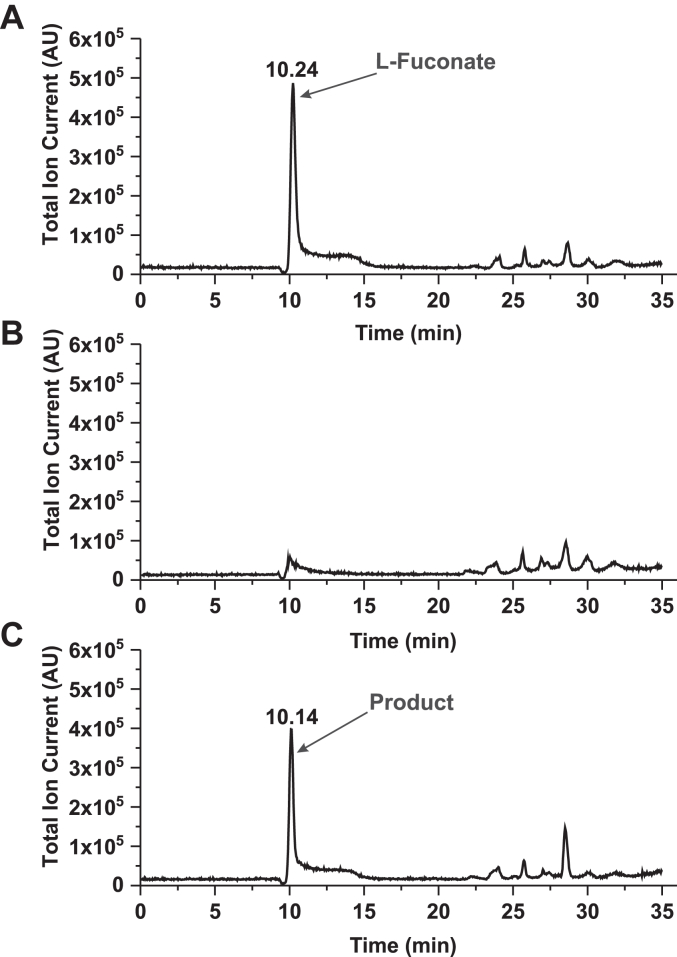
**IC-MS analysis of the product formed by human HSD17B14 protein**. Shown are chromatograms of (*A*) a solution of commercial sodium L-fuconate (12.5 nmol) prepared in the reaction mixture for the enzyme assay; (*B*) of deproteinized reaction mixtures obtained during incubation of homogenous recombinant human protein (2.2 μg) with 2 mM L-fucose and 0.75 mM NAD^+^ for 0 min or (*C*) 10 min The identity of all indicated compounds was confirmed by tandem mass spectrometry. The sample processing and chromatographic conditions are described under "Experimental procedures".

**Figure 9:**
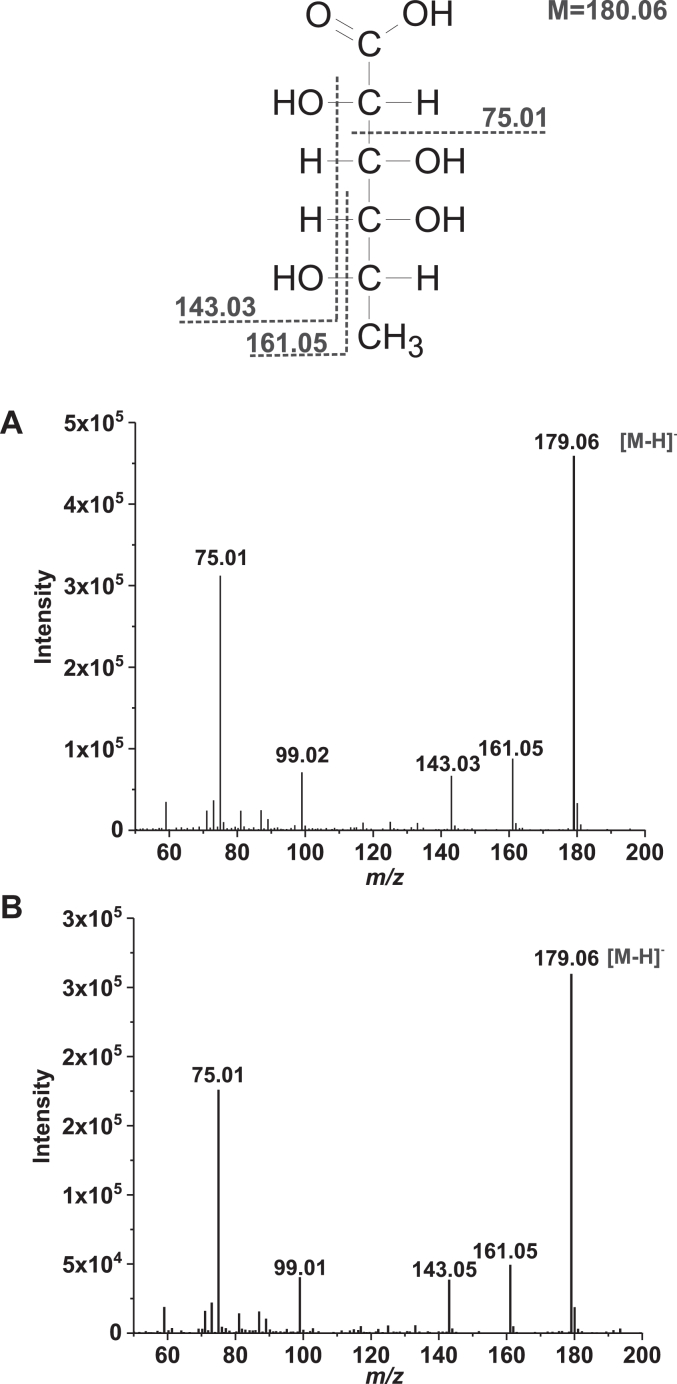
**Q-TOF fragmentation spectra of L-fuconate and the product formed by human HSD17B14 protein.** The homogenous recombinant human enzyme was incubated for 10 min with 2 mM L-fucose and 0.75 mM NAD^+^, and the progress of the reaction was followed spectrophotometrically at λ = 340 nm. The reaction mixture was then deproteinized by adding methanol and acetonitrile (1:1:1), chromatographed on an anion exchange Dionex IonPac AS11HC column, and analyzed by tandem mass spectrometry. Mass spectra, covering the mass range *m/z* 50 to 300, (*A*) of commercial L-fuconate and (*B*) the product biocatalyzed by human HSD17B14 enzyme were acquired. The structure of L-fuconic acid and the assignments of some of its fragment ions are also shown.

Until now, the reaction catalyzed by L-fucose dehydrogenase was thought to produce L-fucono-1,5-lactone, which is unstable and hydrolyzes to L-fuconate, but this view was based on the results of just an elementary chemical analysis (14, 18). To determine the identity of the product of the reaction catalyzed by the recombinant human HSD17B14 enzyme, a standard reaction with L-fucose and NAD^+^ was performed. The reaction was monitored spectrophotometrically and stopped by transferring the reaction mixture to acetonitrile and methanol (1:1:1), followed by precipitated protein removal *via* centrifugation. The clarified supernatant was subjected to an automated ion chromatography and mass spectrometric analysis. Chromatographic analysis of the product revealed its comigration with a commercial standard of L-fuconate (Fig. 8), supporting the identity as the L-fuconate. Analysis of the product by negative electrospray ionization Q-TOF MS indicated a deprotonated parent molecular ion with *m/z* 179 consistent with L-fuconate. The tandem MS analysis of this ion revealed a fragmentation pattern identical to that of the commercial standard of L-fuconate (Fig. 9). Similar results were also obtained for the product formed in the reaction catalyzed by the rabbit enzyme (Figs. S4 and S5).

To confirm the identification of the biocatalyzed product as L-fuconate, an experiment was performed to observe the real-time oxidation of L-fucose using NMR spectroscopy. The experiment employed a minimal amount of enzyme (human HSD17B14, 350 nM) and an excess of substrates (an equimolar mixture of 10 mM L-fucose and NAD^+^). A series of 1D ^1^H datasets was recorded: every 2 min for the first hour, followed by every 10 min for the next 8 h, and then every 8 h for a total of 120 h after the enzyme was added to initiate the reaction. Our analyses of the NMR data were based on previously published signal assignments (15).

**Figure 10:**
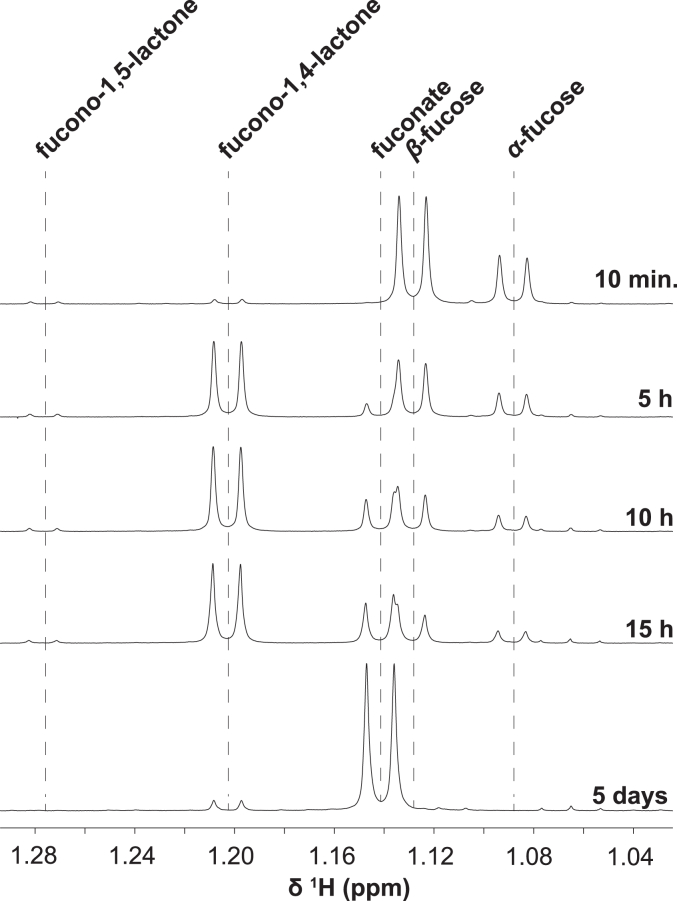
**L-fucose oxidation monitored by**^**1**^**H NMR spectroscopy.** The methyl region of the spectra contains signals from different species formed during the reaction. In solution, L-fucose is a mixture of α- and β-anomers at the approximate ratio of 30:70 and is rapidly oxidized by human HSD17B14 to L-fucono-1,5-lactone. This compound is unstable and quickly converts to L-fucono-1,4-lactone, which in turn hydrolyzes to L-fuconate in a slow nonenzymatic process.

Here, we focused on the resonances of C6-methyl moieties, which proved to be very convenient for elucidating the products of this reaction. At equilibrium, two doublets corresponding to α- and β-anomers of L-fucose at 1.088 and 1.129 ppm, respectively, were identified (Fig. 10, top). Shortly after the reaction was started, two other sets of doublets became apparent: at 1.205 ppm and 1.277, arising from L-fucono-1,4-lactone and L-fucono-1,5-lactone, respectively. Although the L-fucono-1,5-lactone signal remained at a very low but constant level, as the reaction proceeded, the signal intensity of the L-fucono-1,4-lactone methyl group increased quickly. Moreover, a signal of linear L-fuconate appeared a few hours later and reached its maximum intensity after several days of incubation when the entire L-fucose was already consumed. Importantly, the only reagent still present in the reaction mixture, apart from L-fuconate, was L-fucono-1,4-lactone (Fig. 10, bottom). These results clearly indicate that L-fuconate is a side product of the reaction, formed from L-fucono-1,4-lactone through nonenzymatic hydrolysis. Furthermore, L-fucono-1,5-lactone is exceptionally unstable under the reaction conditions and rapidly converts to L-fucono-1,4-lactone. Similar observations have previously been reported for L-fucose oxidation by *Burkholderia* enzymes (15).

We currently hypothesize that the detection of L-fuconate in MS/MS analyses resulted from the spontaneous hydrolysis of the lactone in the ESI source. Therefore, the identification of L-fucono-1,5-lactone and not L-fuconate as the immediate product of the activity of HSD17B14 highlights the importance of NMR analysis for the accurate identification of reaction products.

## Discussion

### Identification and biochemical properties of mammalian L-fucose dehydrogenase

More than 50 years have passed since experiments demonstrated that L-fucose is readily oxidized to CO_2_ in the human body, and the monosaccharide is catabolized into lactate and pyruvate through a specific metabolic pathway that exists in the liver and kidneys of several mammals (10, 12, 13). However, despite these encouraging findings, the biological significance of the pathway remains unclear, and L-fuconate dehydratase, designated as enolase superfamily member 1, is the only mammalian enzyme of the pathway that has been identified so far (20).

Here, we report the identification of rabbit L-fucose dehydrogenase as HSD17B14, disclosing the identity of the enzyme that plausibly triggers the L-fucose degradation in mammalian tissues. This conclusion is supported by the following findings: (i) a multistep purification of the L-fucose dehydrogenase activity from rabbit liver resulted in the identification of the protein HSD17B14 as the only logical candidate for the enzyme activity; (ii) the recombinant rabbit and human HSD17B14 catalyze the NAD^+^-dependent oxidation of L-fucose, yielding L-fucono-1,5-lactone; (iii) a variant of rabbit HSD17B14 harboring a mutation of the key catalytic residue (Y154F) is catalytically inactive; and (iv) the identity of the products made by the recombinant enzymes was confirmed by both mass spectrometry and NMR.

Mammalian HSD17B14 has a typical NAD(H)-binding Rossmann-fold domain in its structure and belongs to the short-chain dehydrogenase/reductase superfamily (22, 28). In humans, enzymes of this group metabolize a wide range of compounds, including steroid hormones, lipids, and xenobiotics (29). HSD17B14 was originally reported as a novel 17-β-hydroxysteroid dehydrogenase that catalyzes estradiol and testosterone oxidation in the C17-hydroxyl group to form estrone and androstenedione, respectively (22). Thus, the enzyme is thought to be functionally similar to HSD17B2 (30). However, human HSD17B14 was reported to be very inefficient in oxidizing steroids, with a k_cat_ value of 0.08 min^−1^ for estradiol and even worse kinetic data for testosterone (22), which was also confirmed in the present study. Such a low turnover number of the enzyme strongly suggests that steroids cannot be physiological substrates for HSD17B14. In fact, it has frequently been speculated that other compounds may be natural substrates for the enzyme (22, 25, 31), since the substrate preference of 17β-hydroxysteroid dehydrogenases seems to be diverse and not restricted to steroids (23).

In this study, we demonstrate that HSD17B14 catalyzes the oxidation of L-fucose to L-fucono-1,4-lactone, which is a slow hydrolyzing compound that forms L-fuconate. However, the initial product of the reaction is L-fucono-1,5-lactone, which is unstable and rapidly transforms to L-fucono-1,4-lactone, as previously shown for bacterial L-fucose dehydrogenase (BmulJ_04919, (15)). These results contradict the previous findings of Schachter *et al.* (14) on the pig enzyme that suggested rapid hydrolysis of L-fuconolactone (probably L-fucono-1,5-lactone) to L-fuconate at the pH of the enzyme reaction. Since we only observed an accumulation of L-fucono-1,4-lactone, and not L-fuconate, during the enzymatic reaction, these differences are probable due to inaccurate analytical methods used by Schachter et al. (14) or contamination of the dehydrogenase preparation with a lactonase that catalyzes the conversion of L-fuconolactones to L-fuconate. In fact, such a lactonase that specifically hydrolyzes either L-fuconolactones has been identified in bacteria (BmulJ_04915, (15)). Therefore, it would be of interest to identify the mammalian counterpart of this enzyme, and we have already initiated that study.

Our results indicate that the substrate specificity of rabbit and human HSD17B14 is identical to that described previously for L-fucose dehydrogenase purified from the pig and rabbit liver (14, 18). The preferred substrate is L-fucose followed by D-arabinose and L-galactose, whereas D-threose and 2-, 3-, and 4-epimers of D-arabinose are oxidized very poorly. This indicates that HSD17B14 preferentially acts on the pyranose form of the sugar and the configuration of hydroxyl groups in C-2 through C-4 is critical for enzymatic activity. The presence of the methyl group at C-6 of L-fucose is apparently not essential for the enzyme; however, its substitution by a hydrogen atom (D-arabinose) or by a hydroxymethyl group yields less effective substrates. More importantly, estradiol is unlikely the physiological substrate as the catalytic efficiency (k_cat_/K_M_) of the rabbit and human enzymes on L-fucose (3081 and 4878 min^−1^ × mM^−1^, respectively) was ≈230- and ≈360-fold higher than that on estradiol (13.6 min^−1^ × mM^−1^) (22).

The negligible activity of rat HSD17B14 on L-fucose and related monosaccharides is consistent with earlier observations, pointing to the lack of substantial L-fucose catabolism in rats (12) and the absence of L-fucose dehydrogenase activity in the liver from this species (13). However, both protein sequence and structural comparisons indicate that the rat enzyme is remarkably similar to human HSD17B14 (*cf.* Figs. 3 and S6*D*). This may suggest that other monosaccharides are preferred substrates for the rat enzyme, and relatively few changes in the HSD17B14 sequence are required to shape its substrate specificity.

In pigs, L-fucose dehydrogenase is active mostly in the liver and kidney, although some minor activity is also detected in other tissues (13). This is in agreement with the tissue distribution of human HSD17B14 protein, whose gene is primarily expressed in proximal tubular cells and hepatocytes (Human Protein Atlas), further supporting our identification.

### Role of L-fucose degradation in mammals

In certain bacteria, including *Xanthomonas campestris* and *Burkholderia multivorans*, L-fucose can serve as the sole carbon source. This monosaccharide undergoes an oxidative degradation pathway culminating in the formation of 2,4-diketo-3-deoxy-L-fuconate, which is subsequently hydrolyzed into pyruvate and L-lactate (as depicted in Fig. 1*B*). This same metabolic pathway is evident in the livers and kidneys of pigs, humans, and most likely other mammals, suggesting that L-fucose degradation is an ancient and metabolically significant process. However, its precise function in mammals remains enigmatic. Due to the scarcity of bioavailable L-fucose in the human diet, its role in energy production seems unlikely. Moreover, L-fucose is primarily utilized for glycoprotein synthesis, making it counterintuitive to break down this dietary monosaccharide instead of channeling it towards glycoprotein salvage pathways (*cf.* Fig. 1*A*). The free L-fucose concentration in human serum is rather low, at approximately 1.7 μM (7), and can only rise to 200 μM after excessive oral supplementation with L-fucose (100 mg/kg body weight), exceeding the rate of its rapid systemic degradation (11). These observations suggest that the L-fucose degradation pathway may serve to limit the presence of L-fucose in serum. This hypothesis merits consideration for several reasons. First, L-fucose is a potent inhibitor of sodium-dependent myoinositol transport (32). Rats, which cannot degrade L-fucose, fed high amounts of fucose (20% of total food weight) showed significant axonal atrophy and demyelination attributed to the depletion of nerve myoinositol (33). Second, similar to other monosaccharides, L-fucose can spontaneously and nonenzymatically react with proteins to form a relatively stable ketoamine adduct in a process of glycation (34). This ketoamine adduct is probably capable of forming a closed-ring structure, taking the form of a furanose, as has previously been shown for glucose-glycated albumin (35). One can speculate that the nonenzymatically formed fucofuranosyl proteins might interact with cellular receptors with activity for L-fucosyl-glycans, therefore disrupting the proper biochemical signaling. Third, a low serum concentration of L-fucose prevents its excessive presence in the urine. This may contribute to limit bacterial growth in the urine track and decrease urinary infections.

In conclusion, we have identified the HSD17B14 protein as the mammalian L-fucose dehydrogenase. It catalyzes the oxidation of L-fucose to L-fucono-1,5-lactone, which rapidly converts to L-fucone-1,4-lactone, and not to L-fuconate as previously proposed. This finding establishes HSD17B14 as the initial enzyme of the putative L-fucose degradation pathway in mammals, dispelling the notion of its primary role in steroid metabolism. Notably, the minimal activity of rat HSD17B14 towards L-fucose aligns with the absence of L-fucose metabolism observed in this species. Finally, this work also appeals to the search for the remaining enzymes that contribute to the L-fucose degradation pathway in mammals.

## Experimental procedures

### Materials

Reagents of analytical grade, whenever possible, were from Sigma-Aldrich, Thermo Fisher Scientific, or Carl Roth. L-fucose was purchased from Alfa Aesar (98% purity) or Sigma-Aldrich (≥99% purity), whereas the remaining sugar substrates were purchased from Sigma-Aldrich, Thermo Fisher Scientific, or Biosynth. L-[1–13C]-fucose was from Biosynth. β-Estradiol and sodium L-fuconate came from Sigma-Aldrich and NAD^+^ from BioShop. DEAE-Sepharose FF resin, Superdex 200 16/60 HiLoad, and HisTrap FF crude columns were obtained from Cytiva. Reactive Red 120 Agarose came from Sigma-Aldrich. Vivaspin 500 centrifugal concentrators were from Sartorius. Enzymes and DNA-modifying enzymes were obtained from Thermo Fisher Scientific or A&A Biotechnology. Leupeptin and antipain were purchased from Carl Roth.

### Assay of L-fucose dehydrogenase activity

The enzyme activity was determined by the modified method employed previously (14). Briefly, the enzyme activity was followed spectrophotometrically by measuring the rate of NAD^+^ reduction to NADH, which is accompanied by an increase in absorbance at λ = 340 nm (ε = 6.22 mM^−1^ cm^−1^). The standard reaction mixture (1 ml) contained the following: 30 mM Tris–HCl, pH = 8.0; 1 mM MgCl_2_; 20 mM KCl; 1 mM DTT; 0.5 mM NAD^+^; 2 mM L-fucose. The latter was prepared as a 20 mM solution in 10 mM Tris–HCl; pH = 8.0. The stock solution was then preincubated for 1 h at 37 °C to reach a chemical equilibrium between β-L-fuco- and α-L-fucopiranose (≈70:30) (24, 36). The reaction was started by adding the enzyme preparation and carried out at 37 °C unless otherwise described. The actual concentration of L-fucose in a stock solution was verified spectrophotometrically by using the above-described continuous spectrophotometric assay. The reaction was carried out for approximately 30 min, until the whole substrate was consumed. Substrate specificity of the enzymes was determined in the standard incubation mixture. All reactions were linear for at least 10 min under all studied conditions.

Due to difficulties in measuring low activity rates of the enzymes studied in the presence of β-estradiol by spectrophotometric tests, assays were performed with the use of the Shimadzu RF-5301PC spectrofluorimeter at 25 °C, with constant mixing. Briefly, the reaction mixture (2 ml) contained the following: 30 mM Tris–HCl, pH = 8.0; 1 mM MgCl_2_, 20 mM KCl, 1 mM DTT, 0.5 mM NAD^+^, 5 μM β-estradiol, and 0.01% DMSO derived from β-estradiol solution. Due to a low solubility of β-estradiol in water (25), 5 μM β-estradiol was the maximal concentration of the steroid that could be tested. The reaction was started by adding the enzyme preparation, and the activity was followed by measuring the rate of NAD^+^ reduction to NADH, which is accompanied by an increase in fluorescence (λ_ex_ = 340 nm, λ_em_ = 465 nm) (37). The activity of the recombinant enzymes towards 5 μM L-fucose was measured under the same conditions. Considering the higher sensitivity of the spectrofluorimeter, activity of the recombinant rat HSD17B14 was also assayed using the fluorimetric method.

Kinetic properties of the enzymes were determined in the reaction mixture (1 ml) that contained the following: 30 mM Tris–HCl, pH = 8.0; 1 mM MgCl_2_; 20 mM KCl; 1 mM DTT; 1.5 mM NAD^+^, and variable concentrations of L-fucose (from 0.01 mM to 4 mM). The reaction was started by adding 1.05 to 2.20 μg of the enzyme preparations. Measurements for NAD^+^ were performed in a similar reaction mixture with 2 mM L-fucose and variable concentrations of NAD^+^ (from 0.0025 mM to 1 mM). The initial velocities of the reaction were calculated from the initial linear part of the reaction progress curves.

V_max_, K_M_, and k_cat_ for the dehydrogenase activities of the enzymes studied were calculated with Origin 2020 software (OriginLab, https://www.originlab.com/index.aspx?go=PRODUCTS/Origin) by fitting the data to the Michaelis–Menten model using nonlinear regression. The remaining calculations were performed using Microsoft Office Excel 2007.

All data are presented as mean ± SD of at least three independent experiments.

### Purification of L-fucose dehydrogenase

A single male White New Zealand rabbit weighing approximately 2.5 kg was purchased from the Agricultural Experimental Plant Wilanów – Obory of the Warsaw University of Life Sciences (Poland). The animal was euthanized by a percussive blow to the head (Directive 2010/63/European Union of the European Parliament). Rabbit liver (75 g) was homogenized in a Waring Blender 7011HS (3 cycles × 30 s with a pause of 30 s) with three volumes (w/v) of a buffer consisting of 50 mM Tris–HCl, pH 8.5; 5 mM KCl; 1 mM MgCl_2_; 1 mM DTT; 3 μg/ml leupeptin; 3 μg/ml antipain. The homogenate was centrifuged for 20 min at 11,000*g* at 4 °C. The resulting supernatant (225 ml) was then fractionated between 0% and 5% concentration (w/v) of PEG 4000 (PEG-4000, Sigma-Aldrich). After 10 min incubation on ice, the sample was centrifuged for 20 min at 11,000*g* at 4 °C. The supernatant was again submitted for fractionation with PEG-4000 concentration (w/v) between 5% and 30%. After 10 min incubation on ice, the sample was centrifuged for 20 min at 11,000*g* at 4 °C. The 5 to 30% precipitate was dissolved in 200 ml of homogenization buffer and frozen at −70 °C before purification.

Clarified sample 5 to 30% was applied to a DEAE-Sepharose column (270 ml; Cytiva) equilibrated with buffer A consisting of 50 mM Tris–HCl, pH 8.5; 5 mM KCl; 1 mM MgCl_2_; 1 mM DTT; 2 μg/ml leupeptin, 2 μg/ml antipain. The column was washed with 400 ml of buffer A, developed with a linear NaCl gradient (0–1 M in 560 ml) in buffer A, and fractions (7 ml) were collected. The most active fractions from the DEAE-Sepharose column were pooled (28 ml) and dialyzed into buffer B (100 mM Na^+^ phosphate, pH 5.0; 5 mM KCl; 1 mM MgCl_2_; 1 mM DTT; 2 μg/ml leupeptin; 2 μg/ml antypain; 4 °C, overnight). The dialyzed sample was then centrifuged (15 min, 4 °C, 10,000*g*), and the pellet was discarded, while the supernatant was subjected to further purification. Because of the relatively high activity of the obtained enzyme preparation, only a half volume of the supernatant was taken for further purification steps. NaCl was added to this supernatant to a final concentration of 3 M. The protein precipitate formed was collected by centrifugation (15 min, 4 °C, 10,000*g*) and then dissolved in 3.5 ml of buffer C: 30 mM Tris–HCl, pH 8.0; 20 mM KCl; 1 mM MgCl_2_; 150 mM NaCl; 1 mM DTT; 2 μg/ml leupeptin; 2 μg/ml antypain. Two and a half milliliter of the obtained sample was loaded onto a Superdex 200 16/600 HiLoad column (120 ml; Cytiva) equilibrated with buffer C. The column was developed with 140 ml of buffer C and 1.5 ml fractions were collected. The most active fractions from the Superdex 200 column were pooled (2 ml), and 1.75 ml of the sample was applied to Reactive Red 120 Agarose column (1.5 ml; Sigma-Aldrich). The column was washed first twice with 1 ml of buffer C and then twice again with 1 ml of 300 mM NaCl in buffer C, and fractions (1 ml) were collected. The enzyme did not bind to the resin and flowed out of the column in wash fractions (negative chromatography). Enzymatically active wash fractions from the Reactive Red 120 Agarose were pooled and concentrated ∼12 times using the Vivaspin 500 ultrafiltration unit. All purification steps were performed at 4 °C, and the enzymatic preparation was stored at −70 °C between steps.

### Zymography

After purification, the enzyme preparation was analyzed by zymography (38). This technique allows for the precise localization of the enzyme protein in a polyacrylamide gel under nondenaturing conditions (Native PAGE). Native PAGE is closely related to SDS-PAGE with the difference that neither the gel nor the electrophoresis buffer contains SDS or any other protein denaturation reagent. Also, samples are not denatured prior to loading to the gel; hence, separated proteins are still in their folded state and preserve their enzymatic activity.

L-fucose dehydrogenase was detected after the formation of a colored product (formazan) of its activity in the presence of L-fucose, NAD^+^, nitrotetrazolium blue (NBT; Thermo Fisher Scientific), phenazine methosulfate (Acros Organics) (38). Thirty microliters of 10-fold concentrated sample from the Reactive Red 120 Agarose purification step was supplemented with 10% sucrose and 0.01% bromophenol blue and electrophoresed on a 6% separating polyacryalmide gel (Tris–HCl, pH 8.8) and a Tris-glycine running buffer at 4 °C for 1 h at a constant voltage (180 V). The gel was then incubated in a mixture containing the following: 10 mM Tris–HCl pH 8.0; 30 mM L-fucose; 6 mM NAD^+^; 0.08% NBT; 0.014% phenazine methosulfate for 5 min at 37 °C, rinsed with water (3 times, 5 min), and fixed in 25% ethanol.

### Identification of the rabbit L-fucose dehydrogenase by tandem MS

A preliminary SDS-PAGE analysis of the peak activity fractions from the Reactive Red 120 Agarose purification step revealed the presence of very faint protein bands with a low protein content; consequently, the fractions were 10- and 12-fold concentrated in a Vivaspin-500 ultrafiltration device and reanalyzed by SDS-PAGE and zymography. All visible bands present in the concentrated fractions were cut from the polyacrylamide gels and digested with Trypsin Gold (Promega). In-gel digestions of the peptides were performed as described (39). Peptides were analyzed by nanoUPLC-tandem MS employing Acquity nanoUPLC coupled with a Synapt G2 HDMS Q-TOF mass spectrometer (Waters) fitted with a nanospray source and working in MSˆE mode under default parameters (40). Briefly, the products of in-gel protein digestion were loaded onto a Waters Symmetry C18 trapping column (20 mm × 180 μm) coupled to the Waters BEH130 C18 UPLC column (250 mm × 75 μm). The peptides were eluted from columns in a 1 to 85% gradient of acetonitrile in water (both containing 0.1% formic acid) at a flow rate of 0.3 μl/min. The peptides were directly eluted into the mass spectrometer. Data were acquired and analyzed using MassLynx 4.1 software (Waters) and ProteinLynx Global Server 2.4 software (PLGS; Waters) with a false positive rate of ≤4% as implemented by PLGS software.

The raw data were processed employing Apex3D algorithm implemented in PLGS software with the following settings: (*i*) chromatographic peak width and MS TOF resolution: automatic, (*ii*) lock mass window: 0.25 Da, (*iii*) the low and elevated energy thresholds: 250 and 100 counts, respectively, and (*iv*) the intensity threshold: 1500 counts.

For peptide and protein identification, an MS^∧^E search was performed against a randomized database specified later. The peptide and fragment mass tolerance was set to automatic mode. The minimum number of fragment ion matches required for a peptide was 3; the minimum number of fragment ion matches required for a protein was 7, and the minimum number of peptide matches required for a protein to remain under consideration was set to 1. The primary digest reagent was trypsin, and at most, one missed cleavage was permitted. No secondary reagent was specified. About 250 kDa was set as the maximum protein mass under consideration. The carbamidomethylation of cysteine was the only specified fixed modification, whereas the oxidation of methionine was applied as the variable modification.

To identify L-fucose dehydrogenase, the complete rabbit (*Oryctolagus cuniculus*) reference proteome (38,550 entries) was downloaded on October 21, 2021, from the NCBI Protein database, updated with amino acid sequence of rabbit HSD17B14 as determined in the present work (see below), randomized, and used as a databank for MS/MS software.

### Overexpression and purification of the recombinant rabbit HSD17B14 protein and its mutated form

To identify the complete ORF that encodes the rabbit HSD17B14 enzyme, the sequence of human HSD17B14 protein (NP_057330) was tblasted against rabbit transcript sequences deposited in the Transcriptome Shotgun Assembly Sequence Database (NCBI TSA), resulting in the identification of the searched ORF within the GBCH01173099.1 sequence (GenBank).

Rabbit total RNA was extracted from 220 mg of liver using Total RNA Mini Kit (A&A Biotechnology) according to the manufacturer's instruction. Complementary DNA was synthesized using Revert Aid H Minus reverse transcriptase (Thermo Fisher Scientific), oligo(dT)18 primers, and 2.5 μg of total RNA according to the manufacturer's instruction. The ORF-encoding rabbit HSD17B14 protein was PCR amplified using Pfu DNA polymerase (Thermo Fisher Scientific) and specific 5′ primer containing the initiator codon included in the KpnI site and 3′ primer containing the stop codon flanked by a BamHI site (Table S2). The amplified DNA product of the expected size was digested with the appropriate restriction enzymes and cloned into the mammalian expression system using the pEF6/His B vector (Invitrogen) with a His_6_Tag sequence at the *N*-terminus. The construct was verified by DNA sequencing (Eurofins).

For transfections, HEK293T cells (ECACC) were plated in eight 100 mm cell culture dishes (VWR-Avantor) at a cell density of 2 × 10^6^ cells per plate in Dulbecco's minimal essential medium with high glucose, stable L-glutamine and sodium pyruvate (DMEM, Biowest) supplemented with 100 units/ml penicillin (Biowest), 100 μg/ml streptomycin (Biowest), and 10% (v/v) fetal bovine serum (Biowest), and grown in a humidified incubator under 95% air and 5% CO_2_ atmosphere at 37 °C. After 24 h, each plate was transfected with 7 μg of pEF6/HisB-rbHSD17B14 vector using the TurboFect transfection reagent (Thermo Fisher Scientific) according to the protocol provided by the manufacturer. After 48 h, the culture medium was removed, the cells were washed with 5 ml PBS, and harvested in 1 ml of 30 mM Tris–HCl pH 8.0, containing 20 mM KCl, 1 mM DTT, 1 mM MgCl_2_, 150 mM NaCl, and 2 μg/ml leupeptin and 2 μg/ml antipain. The cells were lysed by freezing in liquid nitrogen and, after thawing and vortexing, the extracts were centrifuged at 4 °C (20,000*g* for 20 min) to remove insoluble material.

For the purification of recombinant rbHSD17B14 protein, the supernatant of the HEK293T lysate was diluted 3-fold with buffer A: 50 mM Tris–HCl pH 7.5, 500 mM NaCl, 10 mM KCl, 30 mM imidazole, 1 mM DTT and applied onto a HisTrap HP column (1 ml, Cytiva) equilibrated with the same buffer. The column was washed with the same buffer as used to dilute the sample, and the retained protein was eluted with a stepwise gradient of imidazole (30–300 mM). The recombinant protein was present in both 150 and 300 mM imidazole fractions. The purity of the preparations eluted with 300 mM imidazole was >98%, as confirmed by SDS-PAGE, while the yield of recombinant protein was 2.25 mg of homogeneous enzyme per 26 mg of soluble HEK293T cell protein. The enzyme preparations were supplemented with 1 mg/ml fatty acid–free BSA (Sigma-Aldrich) and submitted to dialysis against buffer consisting of 30 mM Tris–HCl, pH 8.0, 150 mM NaCl, 20 mM KCl, 1 mM DTT, 1 mM MgCl_2_, 2 μg/ml leupeptin, 2 μg/ml antipain. The purified enzymes were aliquoted and stored at −70 °C.

Mutated form of rbHSD17B14 enzyme (Y154F) was generated by site-directed mutagenesis using a QuikChange II XL kit (Agilent), with pEF6/HisB-rbHSD17B14 plasmid as a template and mutagenic primers: Y154F-rbHSD17B14-S and Y154F-rbHSD17B14-AS (*cf.* Table S2). Mutated form of HSD17B14 was then produced in HEK293T cells and purified using HisTrap FF crude column (5 ml) as described for the WT rabbit enzyme.

### Overexpression and purification of the recombinant human and rat HSD17B14 proteins in a bacterial expression system

The ORF-encoding human enzyme (NM_016246.3) was purchased from DNASU Plasmid Repository (clone ID: HsCD00288523), whereas the ORF coding for rat enzyme (NM_001191111.1) was obtained from GeneScript (clone ID: ORa06939D). The ORFs were PCR amplified using Pfu DNA polymerase and specific primers containing the initiator codon or the stop codon, digested with the restriction enzymes listed in Table S2, and cloned into the pCOLD I vector (Takara Bio), which allows for the production of proteins with an *N*-terminal His6 tag in *E. coli*. Constructs were verified by DNA sequencing (Eurofins).

For protein production, *E. coli* BL21 (DE3) cells were transformated with an appropriate DNA construct, and single colonies were selected to start overnight precultures. Each of four 250 ml portions of LB Broth (1L in total; with 100 mg/ml ampicillin) was inoculated with 2.5 ml of the preculture and incubated (37 °C, 175 rpm) until an absorbance of 0.5 at 600 nm was reached. Next, cultures were placed on ice for 30 min (cold shock) and the IPTG (BioShop) was added to a final concentration of 50 μM or 200 μM to induce human or rat protein expression, respectively. Then, cells were incubated for 16 to 20 h at 14 °C, 175 rpm and harvested by centrifugation (6,000g, 10 min). The cell paste was suspended in lysis buffer consisting of 30 mM Tris–HCl, pH = 8.0, 20 mM KCl, 1 mM MgCl_2_, 1 mM DTT, 1 mM PMSF, 0.125 mg/ml hen egg-white lysozyme (BioShop), and 1000 U of Viscolase (A&A Biotechnology). The cells were lysed by freezing in liquid nitrogen and, after thawing and vortexing, the extracts were centrifuged at 4 °C (20,000*g* for 20 min).

For the purification of recombinant human or rat HSD17B14 proteins, the supernatant of *E. coli* lysate was diluted threefold with buffer A: 50 mM Tris–HCl, pH 7.5, 500 mM NaCl, 10 mM KCl, 30 mM imidazole, 1 mM DTT, and applied onto HisTrap FF crude column (5 ml or 1 ml) equilibrated with the same buffer (A). The column was then washed with 5 to 10 volumes of buffer A, and the retained proteins were eluted with a stepwise gradient of imidazole (30–300 mM). The purity of the preparations eluted with 300 mM imidazole was >93% (rat) and >98% (human), as confirmed by SDS-PAGE, while the yield of recombinant proteins ranged between 1.3 mg (rat) and 4.8 mg (human) of homogeneous protein per 1L of bacterial cell culture. Enzyme preparations were supplemented with 1 mg/ml BSA and submitted to dialysis against buffer consisting of 30 mM Tris–HCl, pH 8.0, 150 mM NaCl, 20 mM KCl, 1 mM DTT, 1 mM MgCl_2_, 2 μg/ml leupeptin, 2 μg/ml antipain. The purified enzymes were aliquoted and stored at −70 °C for further analysis.

### Product analysis

#### Mass spectrometry

To obtain products formed in the reactions catalyzed by recombinant HSD17B14 proteins for MS analysis, 2.1 μg of the homogenous recombinant human enzyme or 4.4 μg of homogenous recombinant rabbit enzyme was incubated in the standard reaction mixture (2 ml) containing 0.75 mM NAD^+^ and 2 mM L-fucose. The reaction was started by the addition of HSD17B14 and followed up spectrophotometrically as described previously. After 0 and 10 min of incubation at 37 °C, 0.3 ml of the reaction mixture was transferred to 600 μl of acetonitrile and methanol (1:1) to stop the reaction and deproteinize the sample. Precipitated protein was removed by centrifugation (12,500*g* for 10 min, 4 °C), and the clear supernatants were analyzed by ion chromatography according to a slightly modified method of Ritter *et al.* (41). L-Fuconate was separated in a gradient mode on the Dionex IonPac AS11HC columns (2 × 250 mm in series, particle size of 5 μm) using the Dionex ICS-3000 Ion Chromatography System and Waters Synapt G2 HDMS Q-TOF mass spectrometer fitted with an electrospray source. The separation was performed using 0.5 mM KOH for the initial 10 min, followed by a linear gradient from 0.5 to 20 mM KOH for 14 min and subsequently, from 20 to 0.5 mM for 1 min at a flow rate of 0.25 ml/min, followed by the column equilibration for a further 11 min under the initial conditions. The conductometric detector monitored the column eluate, followed by the mass spectrometer, operating in negative ESI–MS or ESI–MS/MS mode. The mass spectral data were recorded for *m/z* = 50 to 300 to detect L-fuconate. The ESI source was set at a temperature of 90 °C, the capillary voltage of 2 kV, and the sampling cone voltage of 30 V. The flow rate of the desolvation gas (nitrogen) was 700 l/h, and the desolvation temperature was 130 °C. To confirm the identity of the L-fuconate precursor ion, collision-induced dissociation experiments were run by selecting the target ion (*m/z* 179). The trap collision energy was set to 4.0 eV.

#### Nuclear magnetic resonance

All NMR experiments were conducted using an Agilent spectrometer operating at the proton frequency of 600 MHz, equipped with a room temperature of 5 mm triple resonance HCN probe. All spectra were recorded at 25 °C. Standard ^1^H NMR parameters were employed using a 45-degree pulse of 5 μs, spectral width of 6 kHz, and a relaxation delay of 1 s, while the acquisition time was 2.7 ms. Eight scans were accumulated. Spectra were referenced with DSS signal, processed and analyzed with VnmrJ 4.2, and plotted with MestReNova 12.0.4. Samples used for the NMR experiments were prepared in 50 mM phosphate buffer with 200 mM NaCl, 1 mM MgCl_2_ dissolved in D_2_O (pD 8.0), and contained 10 mM of L-fucose and 10 mM of NAD^+^. The reaction was started by adding 0.35 μM human HSD17B14 when the mutarotation of L-fucose reached equilibrium (40 min after dissolving in the buffer).

### Analytical methods

#### SDS-PAGE

The SDS polyacryalmide gel electrophoresis was performed using a discontinuous one-dimensional electrophoretic system composed of 4% stacking polyacryalmide gel (Tris–HCl, pH 6.8) and 10% separating polyacryalmide gel (Tris–HCl, pH 8.8) and Tris-glycine SDS running buffer (42). The gels were hand-casted in empty mini gel cassettes (1.0 mm thickness, Thermo Fisher Scientific) according to the manufacturer's instructions. Protein samples were dissolved in sample buffer composed of 42 mM Tris–HCl, pH 6,8, 10 mM DTT, 2% SDS, 10% sucrose, 0.01% bromophenol blue, and heated at 80 °C for 5 min. The denatured samples along with the PageRuler prestained protein ladder (Thermo Fisher Scientific) were loaded onto the gels, and electrophoresis was carried out at a constant voltage (180 V), using the XCell SureLock Mini apparatus (Thermo Fisher Scientific). The gels were then stained with PageBlue Protein Staining Solution (Thermo Fisher Scientific) according to the manufacturer's instruction.

#### Protein determination

The protein concentration was determined spectrophotometrically according to Bradford (43) using BSA (Thermo Fisher Scientific) as standard. Alternatively, the concentration of the purified recombinant enzymes in the presence of BSA was determined densitometrically (44). Briefly, protein samples containing increasing amounts of BSA (1–10 μg, Thermo Fisher Scientific) as a standard or purified recombinant enzyme preparations were dissolved in the sample buffer, heated at 80 °C for 5 min, and separated using SDS-PAGE electrophoresis. The gels were then stained with PageBlue Protein Staining Solution (Thermo Fisher Scientific) and analyzed with the use of the ImageJ 1.54g program (National Institutes of Health).

#### Western blot

Recombinant proteins were detected by Western blot analysis, employing a mouse primary antibody against the His_6_ tag (MA1-21315; Thermo Fisher Scientific) diluted in a ratio of 1:1000 in 5% nonfat milk in PBS and a horseradish peroxidase–conjugated goat anti-mouse secondary antibody (A2554, Sigma-Aldrich) diluted in a ratio of 1:20,000 in 1% fatty acid–free BSA (Sigma-Aldrich) in PBS, as previously described (45). The specificity of the antibodies was verified by the vendors. All western-blotting analyses employed chemiluminescence and signal acquisition with Carestream MXBE film, with the pattern of the prestained protein ladder being copied from the blotting membrane onto the film using a set of felt-tip pens.

## Data availability

The MS proteomics data are available at Zenodo repository (https://zenodo.org/) with the data set identifier DOI 10.5281/zenodo.10657428. The complete list of identified proteins and assigned peptides is shown in Supplementary File 1. All other data are contained within the article.

## Supporting information

This article contains supporting information (46, 47, 48).

## Conflict of interest

The authors declare that they have no conflicts of interest with the contents of this article.
