# Supplementary material for: Hydroxysteroid 17-β dehydrogenase 14 (HSD17B14) is an L-fucose dehydrogenase, the initial enzyme of the L-fucose degradation pathway
Source: J Biol Chem. 2024 Jun 27;300(8):107501. doi: 10.1016/j.jbc.2024.107501 (PMC11293516; doi:10.1016/j.jbc.2024.107501)
Supplement: Supplementary Figures [file mmc1.pdf]

Hydroxysteroid 17-β dehydrogenase 14 (HSD17B14) is an L-fucose dehydrogenase, the initial enzyme of the L-fucose degradation pathway

Apolonia Witecka[1], Varvara Kazak[1], Sebastian Kwiatkowski[1,2], Anna Kiersztan[1], Adam K. Jagielski[1], Wiktor Kozminski[3], Rafal Augustyniak[3]*, and Jakub Drozak[1]*

[1]Department of Metabolic Regulation, Institute of Biochemistry, Faculty of Biology, University of Warsaw, Miecznikowa 1, 02-096 Warsaw, Poland

[2]Celon Pharma S.A., Marymoncka 15, 05-152 Kazun Nowy, Poland

[3]Biological and Chemical Research Centre, Faculty of Chemistry, University of Warsaw, Zwirki i Wigury 101, 02-089 Warsaw, Poland

*Corresponding authors:

Rafal Augustyniak

E-mail: rafal.augustyniak@uw.edu.pl

Jakub Drozak

E-mail: j.drozak2@uw.edu.pl

List of supporting informations:

Figures S1 to S6

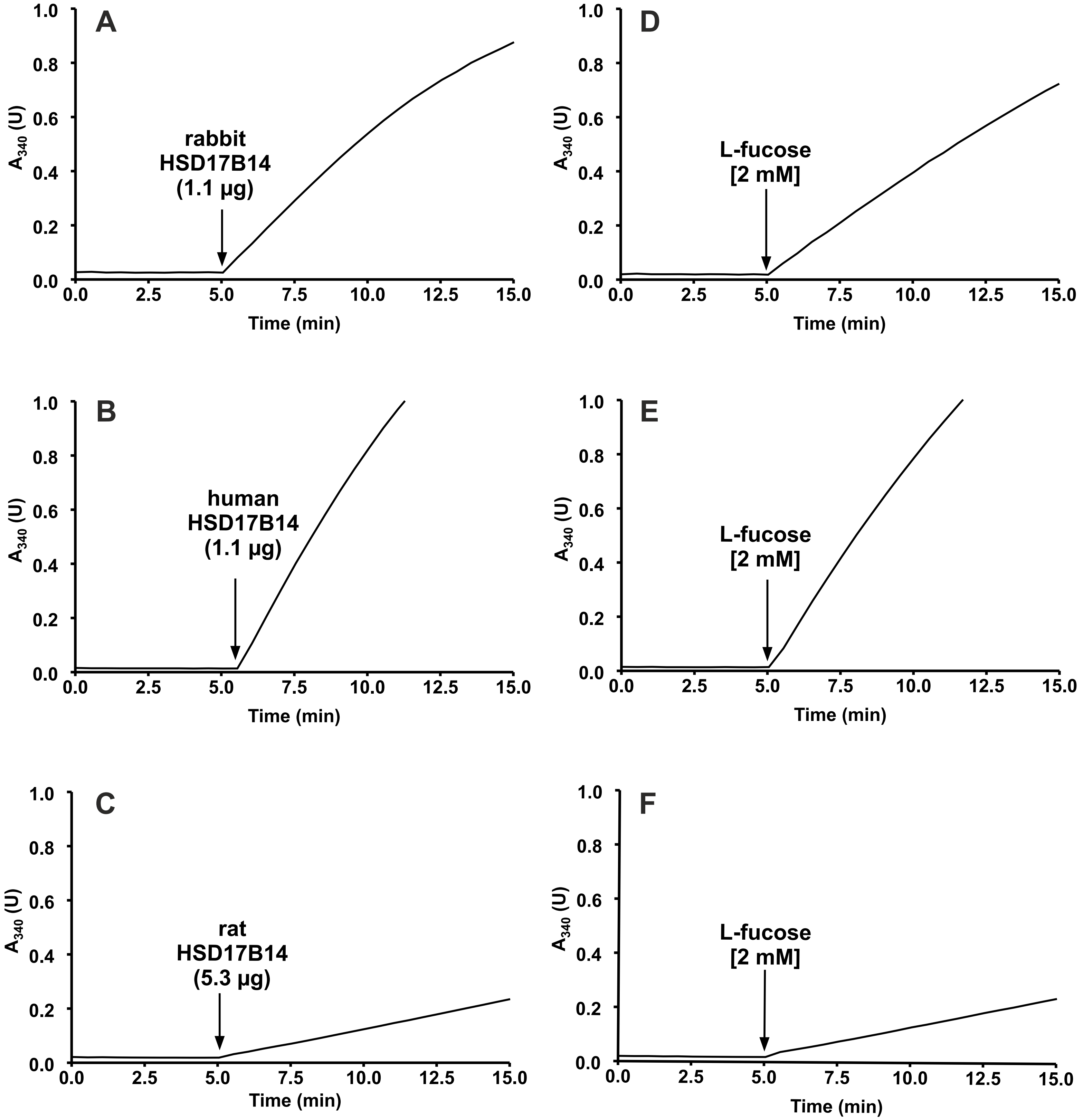

Figure S1. Test of the purified recombinant HSD17B14 activity. The activity of rabbit, human and rat enzymes was followed spectrophotometrically by measuring the conversion of NAD^+ into NADH (= 340 nm). The reactions were performed as described in the "Experimental Procedures" section. Panels A., B., and C. The addition of the indicated amount of the enzyme to the reaction mixture containing L-fucose at 2 mM concentration resulted in a progressive reduction of NAD^+, as indicated by the change in the absorbance (A_{340}). Panels D., E., and F. No reaction was detectable in the presence of enzyme until the substrate (L-fucose) was added.

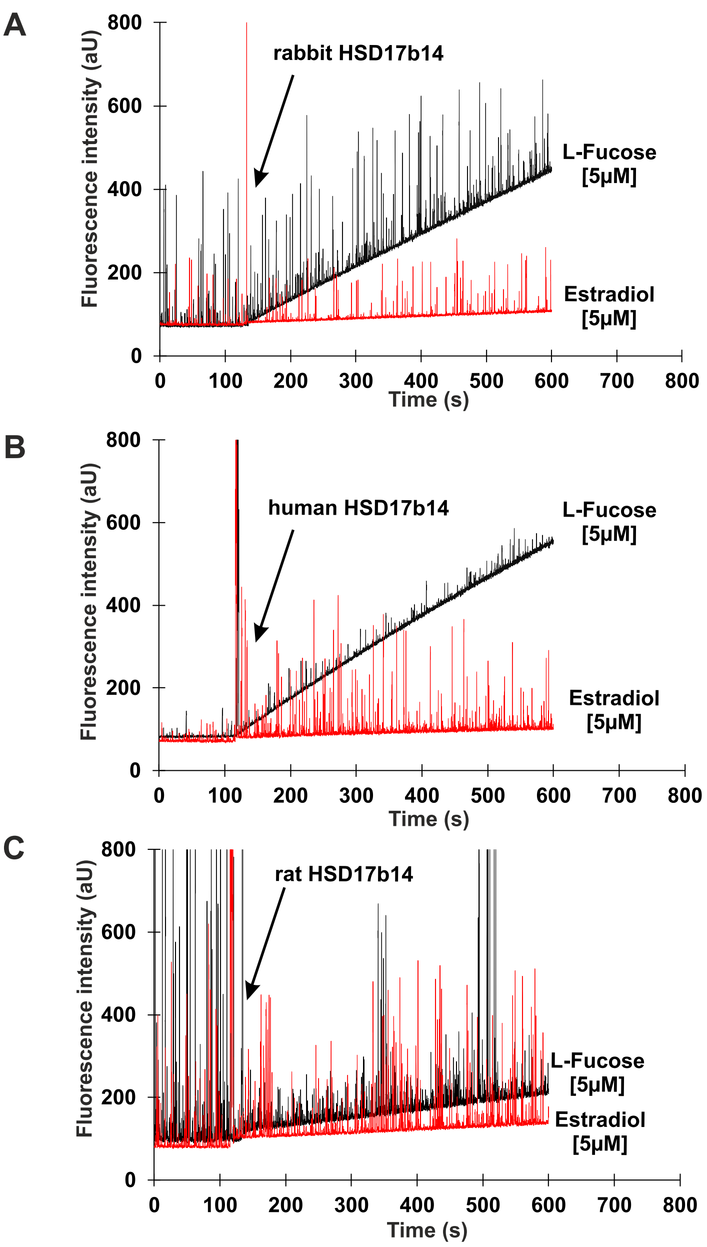

Figure S2. Comparison of HSD17B14 activity in the presence of either L-fucose or estradiol. The activity of purified recombinant. A. rabbit, B. human, and C. rat enzymes was followed fluorometrically by measuring the conversion of NAD^+ to NADH (λ_{ex}=340 nm, λ_{em}=465 nm) in the presence of indicated substrates at 5 µM concentration which corresponds to the maximal aqueous solubility of estradiol [J. Chem. Eng. Data 2006, 51, 3, 879–881]. The reactions were performed as described in "Experimental Procedures". The oxidation of L-fucose was started by adding rabbit (0.26 µg), human (0.26 µg) or rat enzyme (0.13 µg), whereas a noticeable reaction with estradiol required to add 25-fold more enzyme (6.5. 6.5, or 3.3 µg, respectively). The figures show the results of a single representative assay out of three independent measurements.

Note that the fluorescence traces exhibit numerous peaks, likely originating from dust contamination. Dust particles often fluoresce in the blue/green region, and their emission is readily detectable by a spectrofluorimeter operating in high-sensitivity mode. Furthermore, the vigorous and continuous stirring during the measurement can readily disperse even a few dust particles throughout the sample, leading to the appearance of numerous emission peaks in the fluorescence trace.

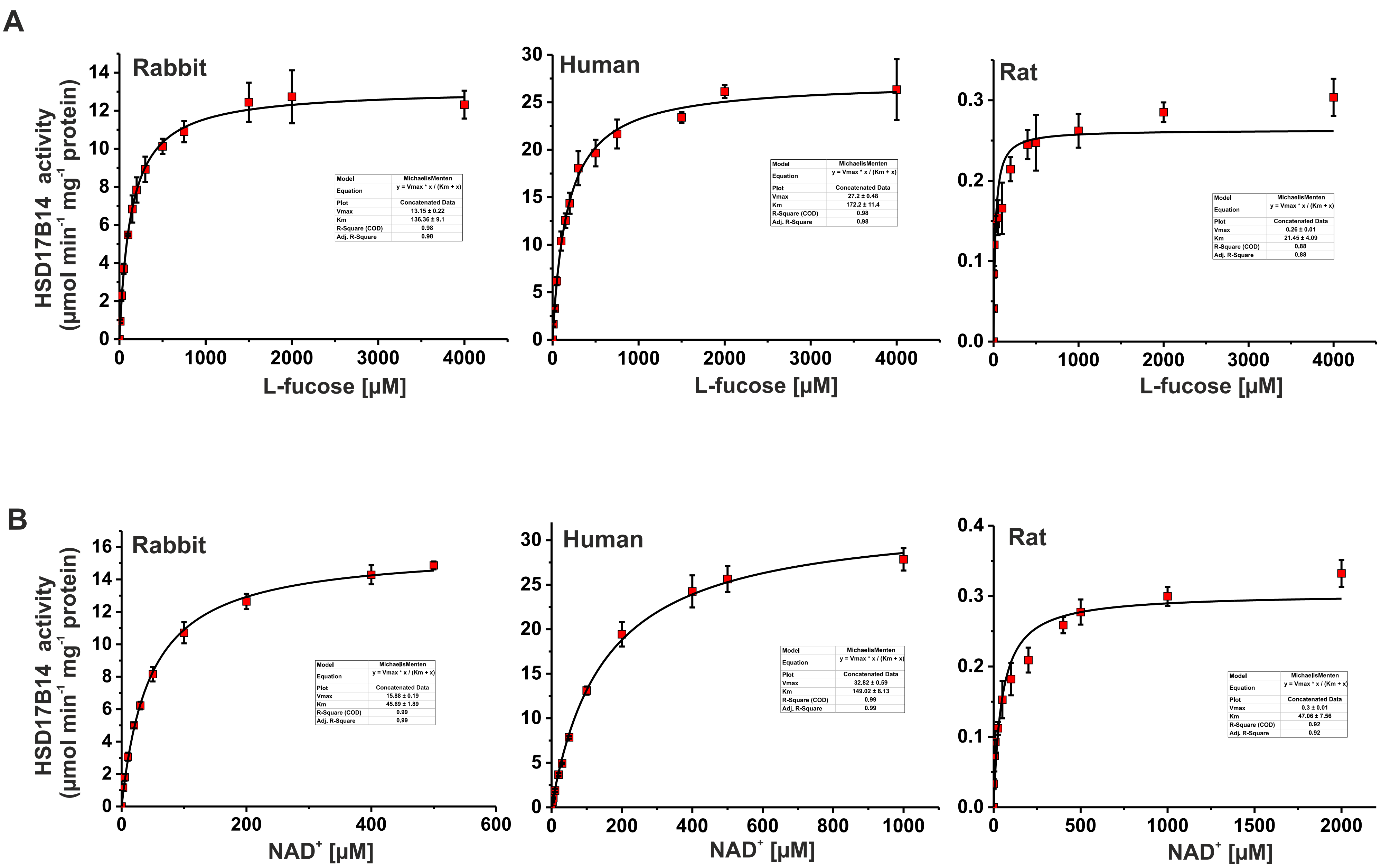

Figure S3. Michaelis-Menten equation curves for the reactions catalyzed by rabbit, human and rat HSD17B14. Michaelis-Menten plots for A. L-fucose and B. NAD^+ are shown. The activity of enzymes was followed spectrophotometrically (human and rabbit HSD17B14) or fluorometrically (rat HSD17B14) by measuring the conversion of NAD^+ into NADH in the presence of L-fucose. The reactions were performed as described in the "Experimental Procedures" section. Values are the means ± SD (error bars) of three independent experiments. The curves were plotted employing Origin 2020 software (OriginLab, USA) and nonlinear regression analysis.

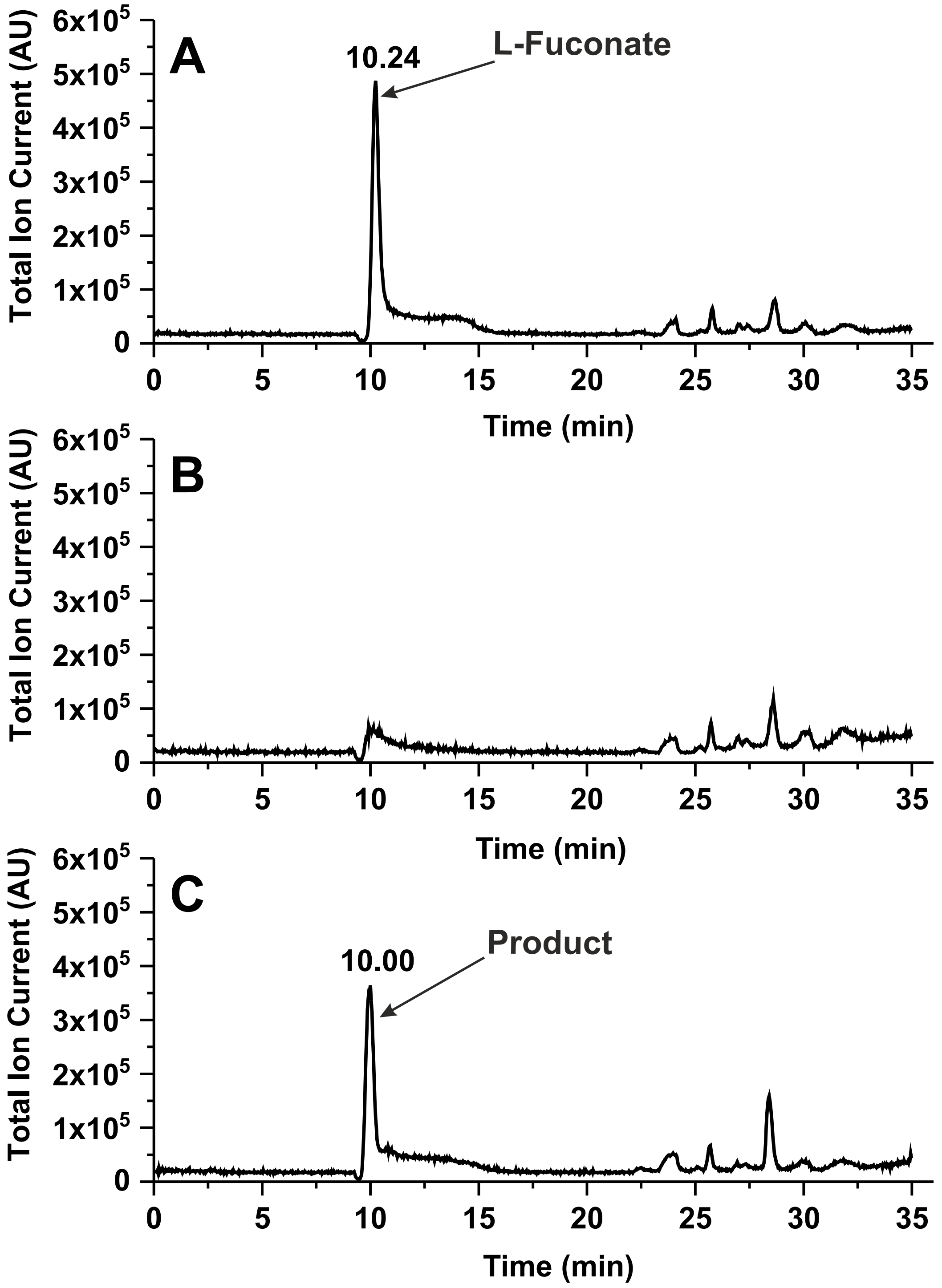

Figure S4. IC-MS analysis of the product formed by rabbit HSD17B14 protein. Shown are chromatograms A. a solution of commercial sodium L-fuconate (12.5 nmol) prepared in the reaction mixture for the enzyme assay; this chromatogram is identical to that shown in Fig. 8A, as chromatograms shown here and in Fig. 8 were obtained in one series of chromatographic analyzes; B. of deproteinized reaction mixtures obtained during incubation of homogenous recombinant rabbit protein (2.2 µg) with 2 mM L-fucose and 0.75 mM NAD^+ for 0 min or C. 10 min. The identity of all indicated compounds was confirmed by tandem mass spectrometry. The sample processing and chromatographic conditions are described under "Experimental Procedures".

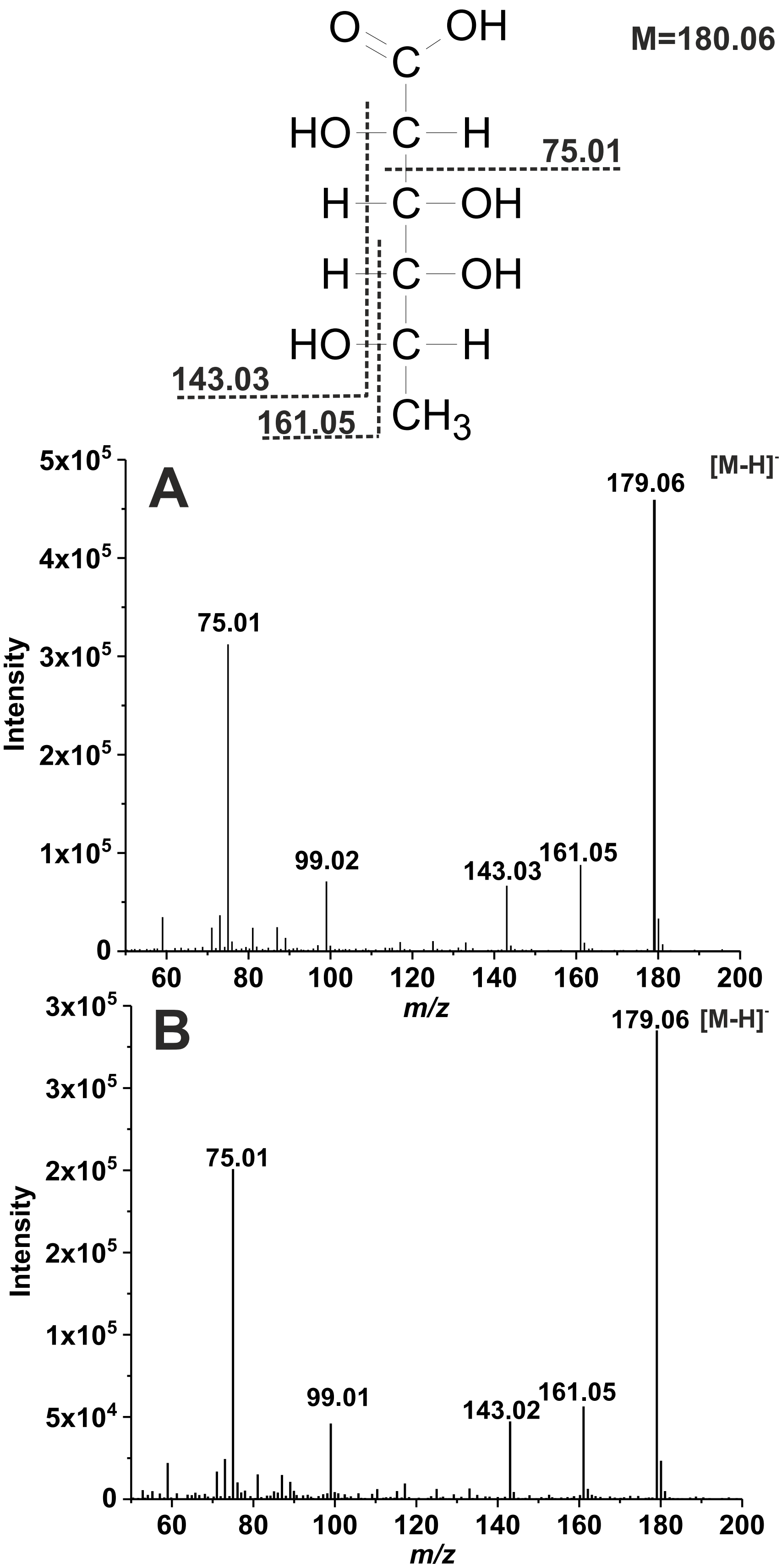

Figure S5. Q-TOF fragmentation spectra of L-fuconate and the product formed by rabbit HSD17B14 protein. The homogenous recombinant rabbit enzyme was incubated for 10 min with 2 mM L-fucose and 0.75 mM NAD^+, and the progress of the reaction was followed spectrophotometrically at λ= 340 nm. The reaction mixture was then deproteinized by adding methanol and acetonitrile (1:1:1), chromatographed on an anion exchange Dionex IonPac AS11 column, and analyzed by tandem mass spectrometry. Mass spectra, covering the mass range *m/z* 50–300, A. of commercial L-fuconate; this spectrum is identical to that shown in Fig. 9A, as the mass spectra shown here and in Fig. 9 were obtained in one series of analyzes, and B. the product biocatalyzed by the rabbit HSD17B14 enzyme were acquired. The structure of L-fuconic acid and the assignments of some of its fragment ions are also shown.

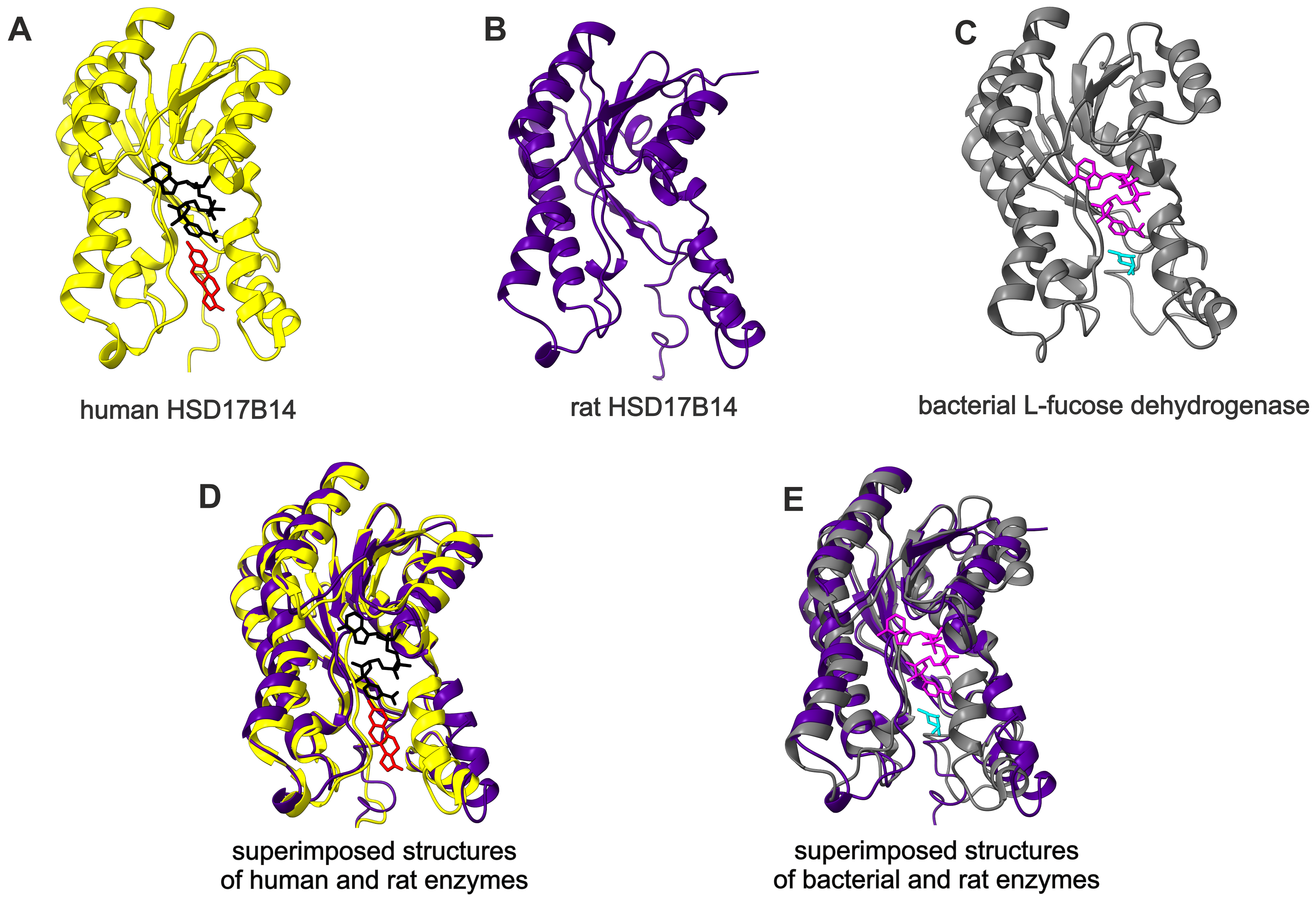

Figure S6. Structural similarity between human and rat HSD17B14 proteins. Ribbon representations of A. human HSD17B14 (PDB: 5hs6), B. rat HSD17B14 predicted from amino acid sequence (NP_001178040.1) with Swiss-Model [Nucleic Acids Res. 2018; 46(W1):W296-W303], C. *Burkholderia multivorans* L-fucose dehydrogenase (PDB: 4gvx), D. the superimposed structure of human and rat HSD17B14, highlighting a similar fold architecture (a root mean square deviation of 1.65 Å). E. the superimposed structure of rat HSD17B14 and bacterial L-fucose dehydrogenase (a root mean square deviation of 3.09 Å). A. Human HSD17B14 is shown in complex with NAD (black sticks) and estrone (red sticks), whereas C. L-fucose dehydrogenase is illustrated with bound NADP (magenta sticks) and L-fucose (cyan sticks). All models were prepared using UCSF ChimeraX [Protein Sci. 2021; 30(1):70-82].
