# Supplementary material for: Hydroxysteroid 17-β dehydrogenase 14 (HSD17B14) is an L-fucose dehydrogenase, the initial enzyme of the L-fucose degradation pathway
Source: J Biol Chem. 2024 Jun 27;300(8):107501. doi: 10.1016/j.jbc.2024.107501 (PMC11293516; doi:10.1016/j.jbc.2024.107501)
Supplement: Supplementary Tables [file mmc3.pdf]

Hydroxysteroid 17-β dehydrogenase 14 (HSD17B14) is an L-fucose dehydrogenase, the initial enzyme of the L-fucose degradation pathway

Apolonia Witecka[1], Varvara Kazak[1], Sebastian Kwiatkowski[1,2], Anna Kiersztan[1], Adam K. Jagielski[1], Wiktor Kozminski[3], Rafal Augustyniak[3]*, and Jakub Drozak[1]*

[1]Department of Metabolic Regulation, Institute of Biochemistry, Faculty of Biology, University of Warsaw, Miecznikowa 1, 02-096 Warsaw, Poland

[2]Celon Pharma S.A., Marymoncka 15, 05-152 Kazun Nowy, Poland

[3]Biological and Chemical Research Centre, Faculty of Chemistry, University of Warsaw, Zwirki i Wigury 101, 02-089 Warsaw, Poland

*Corresponding authors:

Rafal Augustyniak

E-mail: rafal.augustyniak@uw.edu.pl

Jakub Drozak

E-mail: j.drozak2@uw.edu.pl

List of supporting informations:

Tables S1 to S2

Table S1. Proteins identified in gel bands submitted to trypsin digestion and MS/MS analysis.
Identified proteins are listed according to their score as calculated using ProteinLynx Global Server software (PLGS). The molecular weight (mW), sequence coverage, and the number of distinct peptides assigned for each protein are also indicated. Occasional peptide hits corresponding to keratins have not been included.

Gel band	Protein name	NCBI Protein accession number	PLGS score*	Peptides	MW (Da)	Coverage (%)
S1	serotransferrin precursor	NP_001095164.1	3994	26	76563	39.9
S2	urocanate hydratase	XP_002713122.1	1585	14	74317	28.5
	acyl coenzyme A synthetase ACSM1	XP_017197955.1	462	8	74234	20.5
	serotransferrin precursor	NP_001095164.1	123	3	76563	11.2
	stress 70 protein	XP_002710287.1	89	3	73536	5.9
S3	acyl coenzyme A synthetase ACSM1	XP_017197955.1	12981	49	74234	55.8
	liver carboxylesterase 1	NP_001076234.1	9473	41	62251	55.6
	triokinase FMN cyclase	XP_008272642.1	349	5	59345	13
	xaa Pro dipeptidase	XP_017197704.1	166	4	54785	8.9
	acyl coenzyme A synthetase ACSM2B	XP_002711848.1	158	3	63895	8.1
	carboxylesterase 3	XP_002711692.2	105	1	62472	3.3
S4	acyl coenzyme A synthetase ACSM2B	XP_002711848.1	1364	12	63895	33.4
	acyl coenzyme A synthetase ACSM1	XP_017197955.1	1143	12	74234	29.9
	alpha aminoadipic semialdehyde dehydrogenase	XP_002710217.1	966	13	58425	33.8
	alpha 1 antiproteinase F	NP_001075654.1	533	5	45838	19.9
S5	aspartate aminotransferase	XP_002718676.1	2134	13	46429	42.6
	hydroxyacid oxoacid transhydrogenase	XP_008253810.1	555	8	50209	30.9
	4 hydroxyphenylpyruvate dioxygenase	XP_008248783.1	423	7	44798	22.4
	cysteine sulfinic acid decarboxylase	XP_017197373.1	169	5	55035	16.0
	kynureninase	XP_002712183.1	157	4	52462	11.4
	sedoheptulokinase	XP_002718914.1	117	5	51082	11.1
S6	4 hydroxyphenylpyruvate dioxygenase	XP_008248783.1	8255	41	44798	63.9
	aspartate aminotransferase	XP_002718676.1	6974	27	46429	64.4
	beta ureidopropionase	XP_002722597.1	2635	12	43238	42.7
	fumarylacetoacetase	XP_017194433.1	107	2	46229	5.7
S7	beta ureidopropionase	XP_002722597.1	8977	30	43238	52.9
	4 hydroxyphenylpyruvate dioxygenase	XP_008248783.1	1188	12	44798	37.7
	serine pyruvate aminotransferase	NP_001075778.1	527	6	43120	19.1
	acyl coenzyme A synthetase ACSM1	XP_017197955.1	168	5	74234	9.6

S8	Rabbit HSD17B14 protein[#]	Not available	18145	29	28305	75.6
	branched chain amino acid aminotransferase	XP_008250588.1	10832	19	65967	24.4
S9	Rabbit HSD17B14 protein[#]	Not available	11363	16	28305	55.2
	branched chain amino acid aminotransferase	XP_008250588.1	6048	9	65967	16.4
	4 hydroxy 2 oxoglutarate aldolase	XP_002718623.1	5280	10	35258	42.5
	S formylglutathione hydrolase	XP_002720762.1	4552	9	31289	46.1
	ketohexokinase	XP_002710008.1	3052	8	32642	25.8
	caspase 3	XP_008272146.1	232	3	31717	11.5
	omega amidase NIT2	XP_008265263.1	191	5	31298	25.8
Z1	Rabbit HSD17B14 protein[#]	Not available	11062	16	28305	44.1
	branched chain amino acid aminotransferase	XP_008250588.1	9530	8	65967	14.7
	4 hydroxyphenylpyruvate dioxygenase	XP_008248783.1	4623	20	44798	48.3
	liver carboxylesterase 1	NP_001076234.1	3628	21	62251	45.0
	acyl coenzyme A synthetase ACSM1	XP_017197955.1	545	10	74234	22.4

*PLGS score is calculated by the Protein Lynx Global Server (version 2.4) software using a Monte Carlo algorithm to analyze all acquired mass spectral data and is a statistical measure of the accuracy of assignation. A higher score implies greater confidence in protein identity.

[#]HSD17B14 was not found with the available rabbit proteome data base, but only after the latter had been updated with the rabbit HSD17B14 sequence (see main text).

The complete list of identified proteins and assigned peptides is shown in Supplementary File 1.

Table S2. Sequences of primers used for PCR amplification of HSD17B14 ORFs and site-directed mutagenesis experiment

Primer	Sequence	Restriction site	Protein expressed
**WT HSD17B14 proteins**			
rbHSD17B14-S	cttggtacctATGGCCTCCGGGGCCCG	KpnI	*N*-terminal His_6-tagged rbHSD17B14
rbHSD17B14-AS	actggatccTCAGGAAGACAGATCCGGGAC	BamHI	*N*-terminal His_6-tagged rbHSD17B14
hHSD17B14-S	ctcggtaccATGGCTACGGGAACGCGC	KpnI	*N*-terminal His_6-tagged hHSD17B14
hHSD17B14-AS	cttgaattctCAGGAAGGGATATCGGGG	EcoRI	*N*-terminal His_6-tagged hHSD17B14
rHSD17B14-S	aggcatatgATGGCTGCAGTAGGCCGAT	NdeI	*N*-terminal His_6-tagged rHSD17B14
rHSD17B14-AS	tccctcgagTTATGGTGGGAGAATGGGGA	XhoI	*N*-terminal His_6-tagged rHSD17B14
**Site-directed mutagenesis of the rbHSD17B14 protein**			
Y154F-rbHSD17B14-S	AGGGGGTTCCG**TTC**GCGGCCACCAAG	N/A	*N*-terminal His_6-tagged mutated form of rbHSD17B14
Y154F-rbHSD17B14-AS	CTTGGTGGCCGC**GAA**CGGAACCCCCT	N/A	*N*-terminal His_6-tagged mutated form of rbHSD17B14

The nucleotides corresponding to the coding sequences are in capital letters, restriction sites are underlined, and mutated codons are shown in boldface type.

Abbreviation: N/A, not applicable.
